# Efficacy of acupuncture combined with active exercise training in improving pain and function of knee osteoarthritis individuals: a systematic review and meta-analysis

**DOI:** 10.1186/s13018-023-04403-2

**Published:** 2023-12-02

**Authors:** Jia Chen, Hong Guo, Juanhong Pan, Hongpeng Li, Yongshen Wang, Zhixiang Liu, Yulong Xie, Song Jin

**Affiliations:** 1 School of Health Preservation and Rehabilitation, Chengdu University of Traditional Chinese Medicine, Chengdu, Sichuan China; 2 Rehabilitation Department, Hospital of Chengdu University of Traditional Chinese Medicine, No.39, 12 Bridge Road, Jinniu District, Chengdu, 610000 Sichuan China; 3 School of Medical and Life Sciences, Chengdu University of Traditional Chinese Medicine, Chengdu, Sichuan China

**Keywords:** Acupuncture, Active exercise training, Knee osteoarthritis, Systematic review, Meta-analysis

## Abstract

**Objective:**

To conduct a systematic review and meta-analysis to investigate the clinical efficacy of acupuncture combined with active exercise training in improving pain and function of knee osteoarthritis (KOA) individuals.

**Data sources:**

PubMed, EMBASE, The Cochrane Library, Web of Science, China National Knowledge Infrastructure, Wan Fang Data, Technology Periodical Database and China Biology Medicine were searched from their inceptions to April 5, 2023.

**Review methods:**

We analyzed trials of acupuncture combined with active exercise training for KOA. The included studies were of high quality (Jadad ≥ 4) and RCTs. Study selection, data extraction, risk of bias and quality assessment were independently performed by two reviewers. We performed systematic analyses based on different outcome measures, including total efficiency rate, visual analogue scale (VAS), the Western Ontario and Mcmaster Universities Osteoarthritis Index (WOMAC), the Lysholm Knee Scale (LKS) and range of motion (ROM). We used Review Manager 5.3 and Stata/MP 14.0 to analyze the data. And it was verified by trial sequence analysis (TSA). If *I*^*2*^ > 50% and *p* < 0.05, we performed sensitivity analysis and subgroup analysis to find the source of heterogeneity. Publication bias was studied by funnel plot and Egger’s test was used to verify it.

**Results:**

Full 11 high-quality studies (Jadad ≥ 4) including 774 KOA individuals were included in this review for meta-analysis. The results showed that acupuncture combined with active exercise training (combined group) was superior to the acupuncture group in improving the total effective rate [RR = 1.13, 95%CI (1.05, 1.22), *I*^*2*^ = 0%, *P* = 0.70], reducing the pain level (VAS) [MD = − 0.74, 95%CI (− 1.04,  − 0.43),* I*^*2*^ = 68%, *P* < 0.05], improving knee joint function (WOMAC) [MD =  − 6.97, 95%CI (− 10.74, − 3.19), *I*^*2*^ = 76%,* P* < 0.05] and improving joint range of motion (ROM) [MD = 6.25, 95%CI (2.37, 10.04), *I*^*2*^ = 0%, *P* = 0.71]. Similarly, the combined group showed significant improvements in the total effective rate [RR = 1.31, 95% CI (1.18, 1.47), *I*^*2*^ = 48%,* P* = 0.10], pain (VAS) [MD = 1.42, 95% CI (− 1.85, − 1.00), *I*^*2*^ = 65%,* P* = 0.02] and knee function (WOMAC) [MD = 7.05, 95% CI (− 11.43, − 2.66), *I*^*2*^ = 86%, *P* < 0.05] compared with the non-acupuncture group.

**Conclusion:**

The combined effect of all studies showed significant benefits of acupuncture combined with active exercise training in improving the total effective rate, reducing pain, promoting recovery of knee function and expanding range of motion. However, some evaluation indicators are highly subjective and need to be further confirmed by more objective and evidence-based high-quality RCTs in future.

*Systematic Review Registration:* [PROSPERO], identifier [No. CRD42023425823].

**Supplementary Information:**

The online version contains supplementary material available at 10.1186/s13018-023-04403-2.

## Introduction

Knee osteoarthritis (KOA) is the most common degenerative joint disease that can cause joint pain, swelling, stiffness, limited mobility and even disability [1]. The overall prevalence of primary osteoarthritis in people over 40 years old in China is as high as 46.3% and 62.2% in people over 60 years old [2], of which KOA accounts for more than 90% of osteoarthritis. The pathological manifestations of KOA are mainly cartilage fibrosis, softening, ulcer formation and deletion, subchondral osteosclerosis, osteophyte formation and synovitis [3], which seriously affect people’s quality of life. The prevalence of KOA is increasing year by year, and the medical cost is huge [4], bringing a heavy burden to families and society [5]. The etiology of KOA is complex, the pathogenesis is still not clear [6], and studies [7, 8] believe that the occurrence of KOA may be related to age, gender, BMI, heredity, occupation and other factors. At present, the main clinical treatments include surgical treatment, non-steroidal drug treatment, physical therapy, exercise therapy, etc. [8]. However, patients have a hard time reconciling the high cost of surgical treatment, and anxiety is often witnessed in such cases. And drug treatment often brings digestive side effects. One study [9] proposed that acupuncture in the treatment of KOA could play a role in reducing inflammation and analgesia, promoting blood circulation, improving microcirculation, so as to delay the progression of the disease. The core of exercise therapy is the active participation of patients. Proper exercise can improve the function of knee joint, reduce pain and improve the quality of life of patients [10]. In addition, exercise therapy can also reduce the loss of bone density and prevent osteoporosis that may be secondary to KOA. Therefore, acupuncture and exercise therapy have become important treatment methods for KOA. Studies [11, 12] have reported that commonly used acupuncture treatment methods mainly include traditional milliacupuncture, electroacupuncture, floating acupuncture, etc. Exercise therapy includes knee joint activity and function exercises, and traditional Chinese sports such as “Yijin Jing” and “Baduan Jin.” Acupuncture and active exercise training alone are effective in treating KOA, and there is relevant literature that has been published to confirm it [13–15], so it is necessary to improve the inclusion and exclusion criteria to further explore the efficacy of acupuncture combined with active exercise training on KOA, and whether it is better than acupuncture alone or exercise therapy alone, so as to derive a more effective treatment for KOA.

## Methods

The meta-analysis was registered on the International Prospective Register of Systematic Reviews (PROSPERO registration number: CRD42023425823). We also reported conforming to the Preferred Reporting Items for Systematic Reviews and Meta-analyses Statement criteria (PRISMA 2020) (Additional file 1).

## Search strategy

Two reviewers (J.Ch. and H.G.) independently searched PubMed, EMBASE, Web of science, the Cochrane Library, China National Knowledge Infrastructure (CNKI), Technology Periodical Database (VIP), Wan Fang Data and China Biology Medicine (CBM) from the earliest available date until April 5, 2023. Following keywords and their varies were used: acupuncture (AT), active exercise training (exercise), knee osteoarthritis (KOA) and randomized controlled trials (RCTs). The language was restricted to Chinese and English. The full search strategies for all databases are shown in Additional file 2.

## Inclusion criteria

After our review of relevant studies, based on the PICOS frameworks (population, intervention, comparison, outcome and study), the inclusion criteria for this review are as follows: (1) Population: Patients with knee osteoarthritis, regardless of gender, age, race, nationality, duration of disease, excluding patients who had knee surgery. (2) Intervention: Treatment measures in the experimental group included only acupuncture + exercise therapy, or acupuncture + exercise therapy in addition to the control group. Here, acupuncture refers to a separate stabbing method, which does not include moxibustion. And exercise therapy is active exercise training, not passive activities such as massage. (3) Comparison: The control group was treated with acupuncture or exercise therapy or western medicine or exercise therapy combined with western medicine. (4) Outcome: Outcomes must include total efficiency rate and Visual Analogue Scale (VAS) and either the Western Ontario and Mcmaster Universities Osteoarthritis Index (WOMAC) or Lysholm Knee Scale (LKS). Range of motion (ROM) can also be included. (5) Study: High-quality (Jadad ≥ 4) RCTs, published in English or Chinese.

## Exclusion criteria

The exclusion criteria for this review are as follows: (1) the experimental group that received treatments other than acupuncture and exercise therapy, such as moxibustion and Tuina massage; (2) non-RCTs; (3) low-quality article (Jadad < 4); and (4) unable to get full text or incomplete article data.

## Data extraction

Two reviewers (J.Ch. and Y.S.W.) screened the studies and collected the data independently according to the inclusion and exclusion criteria. The information of author, publication year, demographics of participants, intervention, treatment frequency and times, duration, outcomes and Jadad score were recorded. All studies were managed with Endnote X9. Disagreements were resolved by discussion or umpired with a third reviewer (Z.X.L.).

## Quality and risk-of-bias assessment

We assessed the quality of the studies using an improved Jadad scale (0–3: low quality, 4–7: high quality), and only studies with high quality (Jadad ≥ 4) were included. The scores were given independently by two reviewers (J.C. and Y.S.W.). If the results were inconsistent, they were discussed with a third reviewer (Z.X.L.).

Two reviewers (J.C. and Y.S.W.) also separately evaluated the risk of bias. The evaluation was based on the Cochrane Handbook for Systematic Review of Interventions, edition 5.3. Items include: (1) random sequence generation (selection bias); (2) allocation concealment (selection bias); (3) blinding of participants and personnel (performance bias); (4) blinding of outcome assessment (detection bias); (5) incomplete outcome data (attrition bias); (6) selective reporting (reporting bias); and (7) other bias. The quality of the included studies was rated as low/unclear/high risk of bias (low risk of bias as “yes,” high risk of bias as “no,” otherwise was “unclear”). In the case of disagreements, a third reviewer (Z.X.L.) was involved.

## Statistical analysis

We used Review Manager 5.3 software provided by the Cochrane Collaboration for data analyses and presented the final result. For the continuous data, the mean differences (MD) and 95% confidence intervals (CI) were used when outcomes were assessed by the same scale. *I*^*2*^ statistical tests were adopted to assess the heterogeneity among studies. A fixed-effects model was applied to combine the data if the* I*^*2*^ < 50% and *p* > 0.05. If *I*^*2*^ > 50% and *p* < 0.05 implies high heterogeneity, a random-effects model was used for meta-analysis and subgroup analysis or sensitivity analysis was considered to determine the source of heterogeneity. The total efficiency rate is dichotomous data and categorized into two levels ((1) effective and (2) inefficacious). The total efficiency rate means the percentage of the total number of participants categorized in the first two levels. In addition, we used funnel plot to explore publication bias, and then, Stata/MP 14.0 was used to perform Egger’s test on the funnel plot to verify whether publication bias existed.

## Trial sequential analysis

Meta-analysis usually requires multiple tests, and random errors that may sometimes lead to false significant results when data are accumulated, and the increased frequency of statistical tests in a meta-analysis increases the possibility of reporting such results [16]. However, trial sequential analysis (TSA) overcomes the shortcomings of classical meta-analysis and corrects for the chances of type *I* error [17].

TSA.0.9.5.10 beta was used for sequential analyses. If the Z-curve exceeds the traditional boundary but does not cross the TSA boundary, it suggests that a false positive error may be made. If it intersects the TSA boundary, it suggests that the meta-analysis results are robust, even if the RIS is not reached. The Z-curve did not intersect with the traditional cut-off value and the TSA cut-off value, and the positive or negative conclusion could not be drawn. The Z-curve intersects the null line, indicating no significance [18]. We set a two-sided 5% type* I* error risk (α) and 20% type *II* error risk (β) to calculate the amount of information needed, with a 20% relative risk (RRR) reduction and a control event rate derived from data from the meta-analysis.

## Certainty of the evidence

The Grading of Recommendations Assessment, Development and Evaluation (GRADE) system was used to assess the certainty of the evidence of each outcome. Each outcome was evaluated from the following five aspects: limitations, inconsistency, indirectness, imprecision and publication bias. The certainty of the evidence was categorized as “high,” “moderate,” “low,” or “very low.”

## Result

### Selection and inclusion of studies

**Fig. 1:**
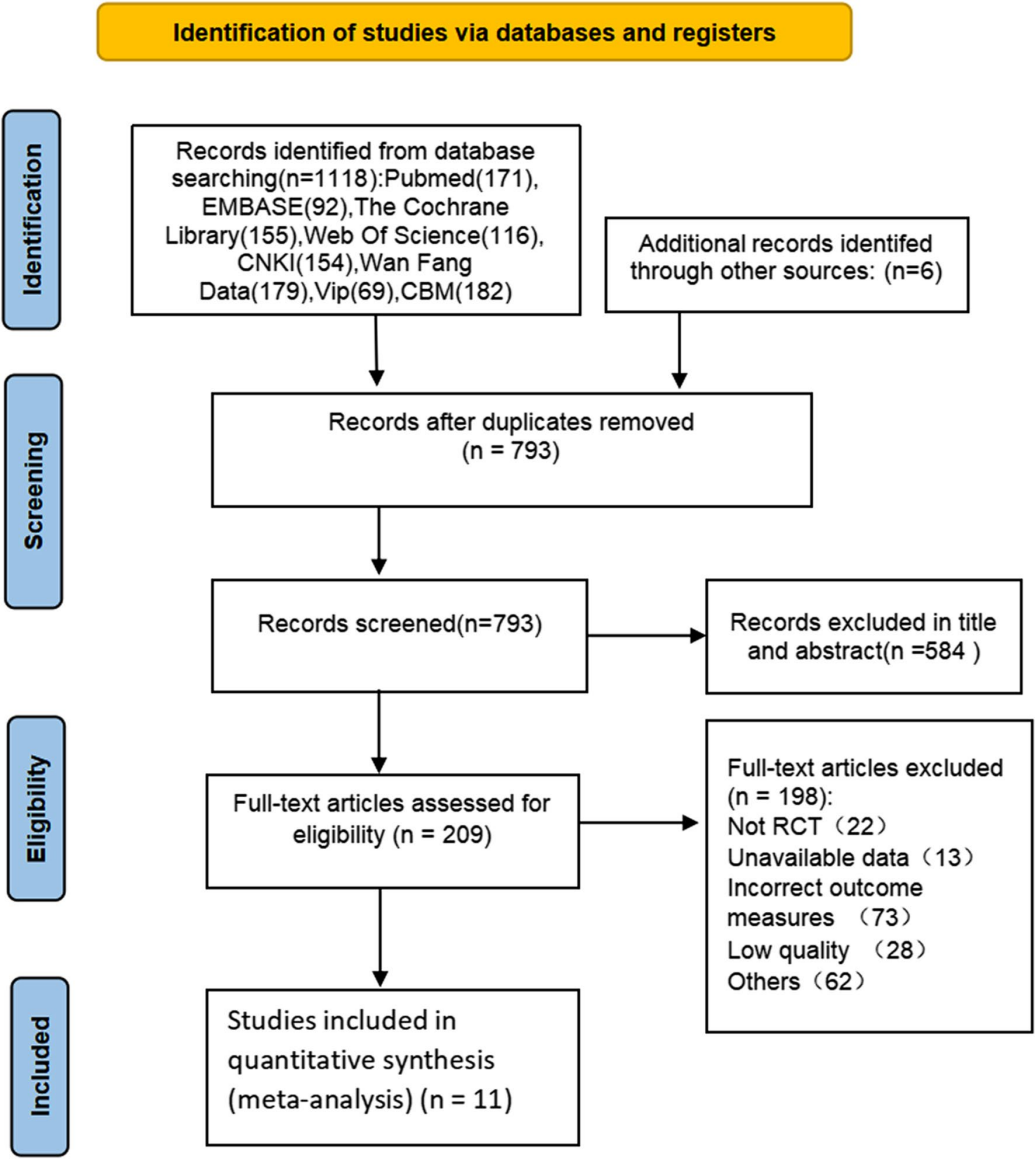
Flowchart of the selection process

A total of 1,124 studies were initially screened (PubMed = 171, EMBASE = 92, The Cochrane Library = 155, Web of Science = 116, CNKI = 154, Wan Fang Data = 179, Vip = 69, CBM = 182, other = 6). After primary searches from the databases, 1124 studies were screened. After duplicates removed, reading the titles and abstracts, 915 studies were excluded. Full texts of 209 studies were retrieved, and 198 studies were excluded with reasons listed as the following: not RCT (n = 22), unavailable data(n = 13), incorrect outcome measures (n = 73), low quality (n = 28) and others (n = 62). In the end, 11 RCTs were included, and 9 were written by Chinese in Chinese [9, 19–26], 2 of which were written by Chinese in English [27, 28]. The detailed screening process is shown in Fig. 1. And the list of excluded records with reasons is provided in Additional file 3.

## Characteristics of included studies

A total of 11 RCTs (one three-arm trial) involving 12 datasets with 774 KOA individuals were included. All of the studies were published between 2011 and 2022 in English or Chinese. The sample size ranged from 56 to 77. All experimental groups received acupuncture combined with active exercise training. Among them, acupuncture included traditional milliacupuncture, electroacupuncture and floating acupuncture, while active exercise training included Yijin Jing, Baduan Jin, walking, functional training around the knee joint. The control groups underwent acupuncture, active exercise training, oral western medicine or exercise combined with western medicine. Characteristics of these studies are shown in Tables 1 and 2.

**Table 1:** Characteristics summary of included studies

References	Age(E/C)	Sample size(E/C)	Intervention		Frequency and times		Duration	Outcomes	Jadad
			E	C	E	C			
Ding [27]	51.7 ± 12.9/59.7 ± 10.4	26/30	Electroacupuncture + Yijinjing	Electroacupuncture	EA:2/w,10 Exercise:3/w,15	EA:2/w,10	5 weeks	^abc^	4
Jin [19]	59.77 ± 8.64/62.67 ± 8.24	30/30	Electroacupuncture + Quadriceps function exercises	Electroacupuncture	EA:3/w,12 Exercise: 3/d,60	EA:3/w,12	4 weeks	^abc^	6
Jin [19]	59.77 ± 8.64/60.40 ± 9.67	30/30	Electroacupuncture + Quadriceps function exercises	Quadriceps function exercises	EA:3/w,12 Exercise: 3/d,60	Exercise: 3/d,60	4 weeks	^abc^	6
Li [28]	57.3 ± 11.6/56.2 ± 12.4	32/32	Acupuncture + Isokinetic eccentric exercise	Isokinetic eccentric exercise + Western medicine	AT:5/w,20 Exercise: 1/d,28	Exercise: 1/d,28	4 weeks	^abd^	4
Lin [20]	49.63 ± 2.72/49.07 ± 3.06	30/30	Acupuncture + Knee function exercises	Western medicine	AT:1/d,14 Exercise: 1/d,14	1/d,14	14 days	^abc^	4
Liu [21]	57.9 ± 8.1/56.0 ± 7.7	38/38	Acupuncture + Isometric exercise	Acupuncture	AT:1/d,20 Exercise: 3/d,60	AT:1/d,20	4 weeks	^abc^	7
Liu [22]	60.10 ± 6.77/61.15 ± 7.08	39/38	Floating acupuncture + Knee joint strength training	Knee joint strength training + Western medicine	FA:1/2d,12 Exercise: 1/2d,12	Exercise: 1/2d,12	4 weeks	^abc^	4
Wang [23]	58.67 ± 5.72/57.17 ± 7.78	30/30	Acupuncture + Guided training	Acupuncture	AT:1/d,20 Exercise:2/d,40	AT:1/d,20	4 weeks	^abce^	6
Wang [24]	56.80 ± 6.45/59.53 ± 7.11	30/30	Acupuncture + Knee flexion and extension exercises	Western medicine	AT:1/d,20 Exercise: 1/d,20	2/d,40	20 days	^abc^	4
Wang [9]	57.77 ± 8.32/57.54 ± 8.20	35/35	Acupuncture + Walking and squatting	Acupuncture	AT:1/d,15 Exercise: 1/d,15	AT:1/d,15	3 weeks	^abcd^	4
Wu [25]	53.79 ± 7.13/53.81 ± 6.15	36/36	Acupuncture + Squatting	Electroacupuncture	AT and Exercise:1/w,4	EA: 3/w,12	4 weeks	^abc^	4
Zheng [26]	57.01 ± 5.59/58.63 ± 5.07	29/30	Acupuncture + Baduanjin	Acupuncture	AT:5/w,20 Exercise: 5/w,20	AT:5/w,20	4 weeks	^abce^	4

^a^Total efficiency rate; ^b^VAS: Visual analogue scale; ^c^WOMAC: The Western Ontario and Mcmaster Universities Osteoarthritis Index; ^d^LKS: Lysholm Knee Scale; ^e^ROM: Range of motion; E/C: Experimental group/control group; EA: Electroacupuncture; AT: Acupuncture treatment; FA: Floating acupuncture; d: day; w: week;

**Table2:** Acupuncture method, selected acupuncture points and exercise mode

References	Acupuncture, acupoints and exercise
Ding [27]	Electroacupuncture, 5 main points,Yanglingquan(GB34),Liangqiu(ST34),
	Zusanli(ST36), Neixiyan(EX-LE4) and Dubi(ST35) + Yijinjing
Jin [19]	Electroacupuncture, 7 main points, Dubi(ST35), Neixiyan(EX-LE4),Xuehai(SP10), Liangqiu(ST34), Yanglingquan(GB34), Zusanli(ST36) and Yinlingquan(SP9) +
	Quadriceps function exercises
Li [28]	Acupuncture, 5 points, Heding (EX-LE2), Dubi (ST35), Neixiyan (EX-LE4), Yinlingquan (SP9) and Yanglingquan (GB34) + Isokinetic eccentric exercise
Lin [20]	Acupuncture, 1 point, Chize(LU5) + Knee function exercises
Liu [21]	Acupuncture, 2 points, Neixiyan(EX-LE4) and Waixiyan(ST35) + Isometric exercise
Liu [22]	Floating acupuncture, Ashi points, + Knee joint strength training
Wang [23]	Acupuncture, 12 points, bilateral Pishu(BL20), bilateral Shenshu(BL23),
	Guanyuan(RN4), Zusanli(ST36), Neixiyan(EX-LE4), Dubi(ST35), Xuehai(SP10), Liangqiu(ST34), Yanglingquan(GB34) and Yinlingquan(SP9) + Guided training
Wang [24]	Acupuncture, 9 points, Neixiyan(EX-LE4), Waixiyan(ST35),Heding(EX-LE2), Liangqiu(ST34), Xuehai(SP10), Yinlingquan (SP9), Yanglingquan (GB34), Zusanli(ST36) and Dazhu(BL11) + Knee flexion and extension exercises
Wang [9]	Acupuncture, 20 points, Dubi (ST35), Neixiyan (EX-LE4),Xiyangguan(GB33),
	Ququan(LR8),Liangqiu(ST34), Xuehai(SP10), Yinlingquan (SP9), Heding(EX-LE2),
	Yanglingquan (GB34),Xiyan(EX-LE5) (All acupuncture points are bilateral) + Walking and squatting
Wu [25]	Acupuncture, 4 points, Xuehai(SP10), Liangqiu(ST34), Yanglingquan(GB34) and Yinlingquan(SP9) + Squatting
Zheng [26]	Acupuncture, 4 points, Xuehai(SP10), Liangqiu(ST34), Neixiyan(EX-LE4), Waixiyan(ST35), Yanglingquan (GB34) and Zusanli(ST36) + Baduanjin

## Methodological quality of included studies

The methodological quality of most included RCTs was generally “high” (Jadad ≥ 4), according to the quality assessment criteria with improved Jadad scale (Table 1). All the trials mentioned the randomized allocation of participants. Selective reporting was generally uncertain in the trials due to the inaccessibility of the trial protocol.

## Risk of bias in studies

**Fig. 2:**
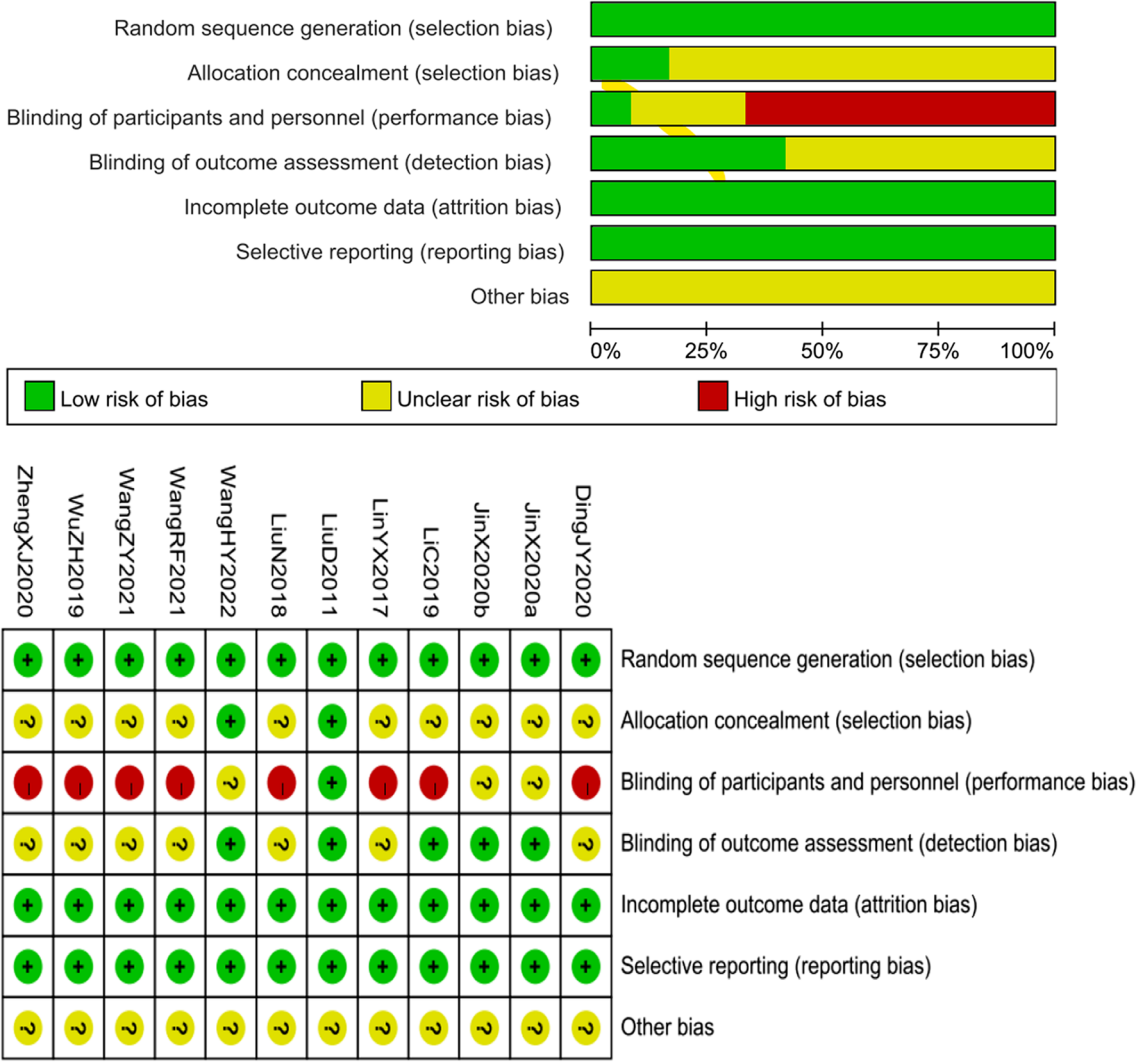
Risk of bias of included studies

The plot of the risk of bias for each included study is shown in Fig. 2. The 12 trials (one three-arm trial) were at low risk. All of 11 studies reported random sequence generation and were assessed as low risk. Ten studies [9, 19, 20, 22, 24–28] were assessed as unclear risk, and two [21, 23] were assessed as low risk in the aspect of allocation concealment. In blinding of participants and personnel, two studies [19, 23] were assessed as unclear risk, one study [21] was assessed as low risk, and eight studies [9, 20, 22, 24–28] were assessed as high risk. Also, seven studies [9, 20, 22, 24–27] were assessed as unclear risk and four studies [19, 21, 23, 28] were assessed as low risk in the blinding of the outcome assessment. Of all these 11 studies were judged to be low risk in incomplete outcome data and selective reporting. Finally, 11 studies were assessed as unclear risk in other bias.

## Publication bias

**Fig. 3:**
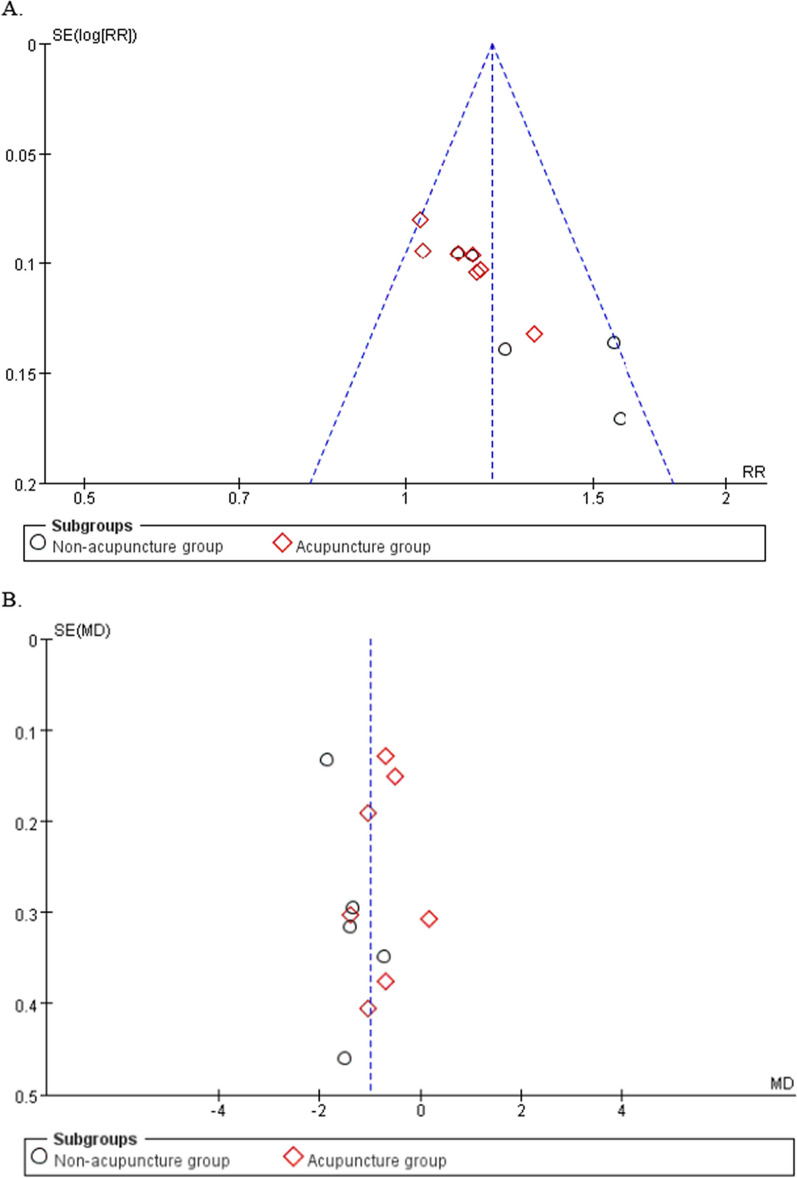
Publication bias of included studies. (A) total efficiency rate. (B) VAS

We first made funnel plots of total efficiency rate and VAS score using Review Manager 5.3, but could not determine whether the funnel plots were symmetrical (Fig. 3A, B). Therefore, we used Stata/MP 14.0 to analyze the publication bias of total efficiency rate and VAS score by Egger’s test. The results showed that the Egger’s test with total effective rate was* p* < 0.05, which may have publication bias. The Egger’s test of VAS score obtained *p* = 0.838 > 0.05, indicating no publication bias.

## Trial sequential analysis

**Fig. 4:**
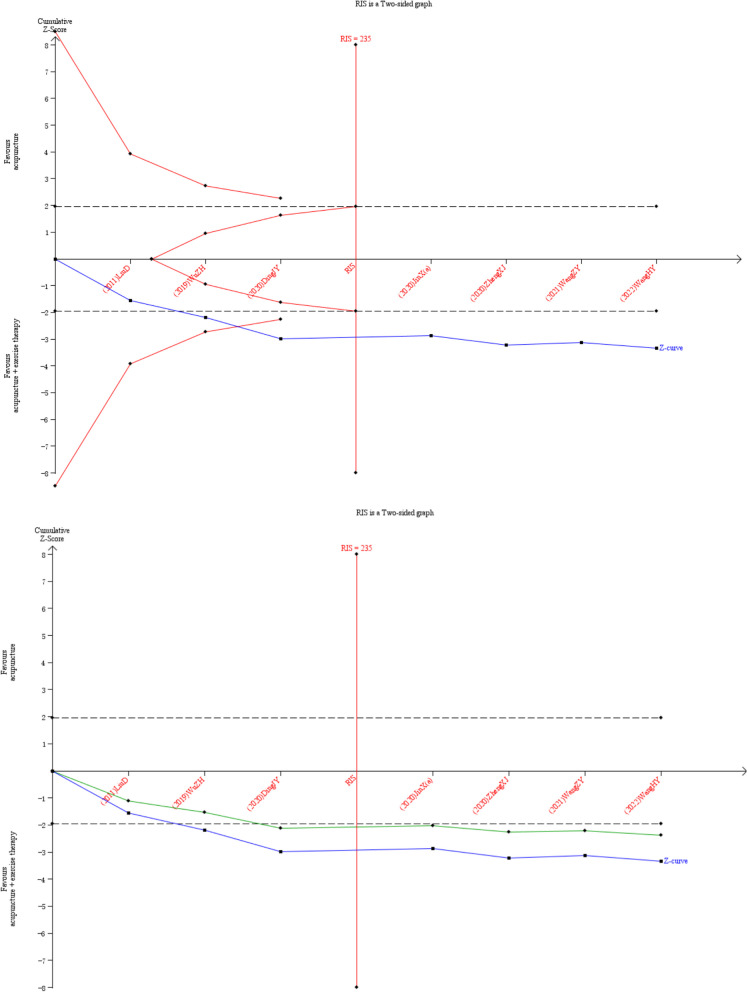
TSA on comparison of combination group versus acupuncture in total efficiency rate. The straight black line represents the conventional statistical boundary of *P* = 0.05. The blue line indicates the cumulative z-score of the meta-analysis. The red line indicates the TSA boundary. The green line represents the Z-curve after the penalty statistic. RIS represents the required size of information

**Fig. 5:**
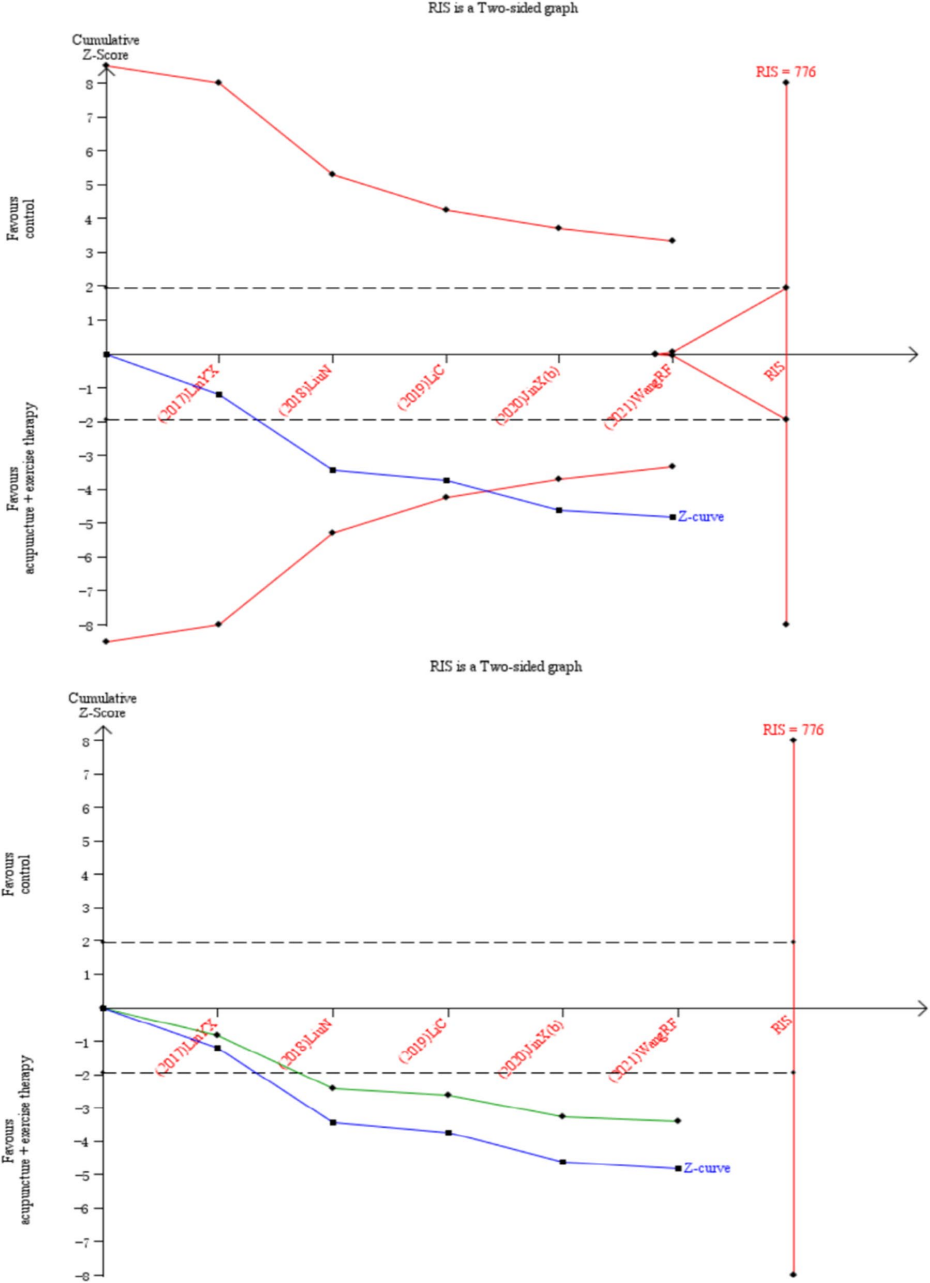
TSA on comparison of combination group versus exercise or western medicine or exercise medicine group in total efficiency rate. The straight black line represents the conventional statistical boundary of *P* = 0.05. The blue line indicates the cumulative z-score of the meta-analysis. The red line indicates the TSA boundary. The green line represents the Z-curve after the penalty statistic. RIS represents the required size of information

Twelve RCTs reported the total efficiency rate, which were analyzed sequentially, with a type *I* error of 5% and a statistical power of 80%. The information axis was set as the cumulative sample size, and the sample size was used as the expected information value (RIS). Figure 4 [9, 19, 21, 23, 25–27] shows that the Z-curve crossed the conventional boundary value and the TSA boundary value, indicating that the results obtained from this meta-analysis were robust and the efficacy of acupuncture combined with active exercise training in the treatment of KOA was positive. Meantime, the penalty curve also exceeded the traditional boundary value and reached the RIS value, which made the meta-analysis result more stable. In Fig. 5 [19, 20, 22, 24, 28], the Z-curve also crossed the conventional boundary value and the TSA boundary value, indicating that the results obtained from this meta-analysis were robust. The penalty curve exceeded the traditional boundary value but did not reach the RIS value. So it needs further research in future.

## Meta-analysis results

Based on various outcome measures (the total efficiency rate, VAS, WOMAC, LKS, ROM), different pooled data from 12 trials were used. The data were divided into stratified analyses according to different interventions of control groups.

### Combination group versus acupuncture group

#### Result of the total efficiency rate

**Fig. 6:**
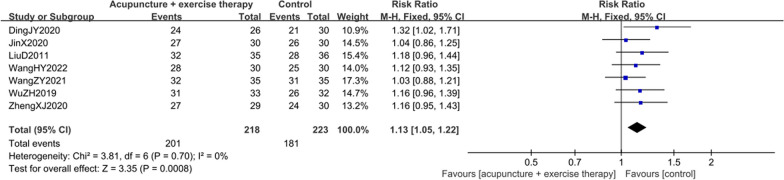
Forest plot of total efficiency rate in comparison with combination group versus acupuncture group

A total of seven studies [9, 19, 21, 23, 25–27] involving 441 KOA individuals compared the total efficiency rate of acupuncture combined with active exercise training and acupuncture for KOA. The results demonstrated that combined treatment was superior to acupuncture in the total efficiency rate [RR = 1.13, 95%CI (1.05, 1.22),* I*^*2*^ = 0%,* P* = 0.70] (Fig. 6).

#### Result of the VAS

**Fig. 7:**
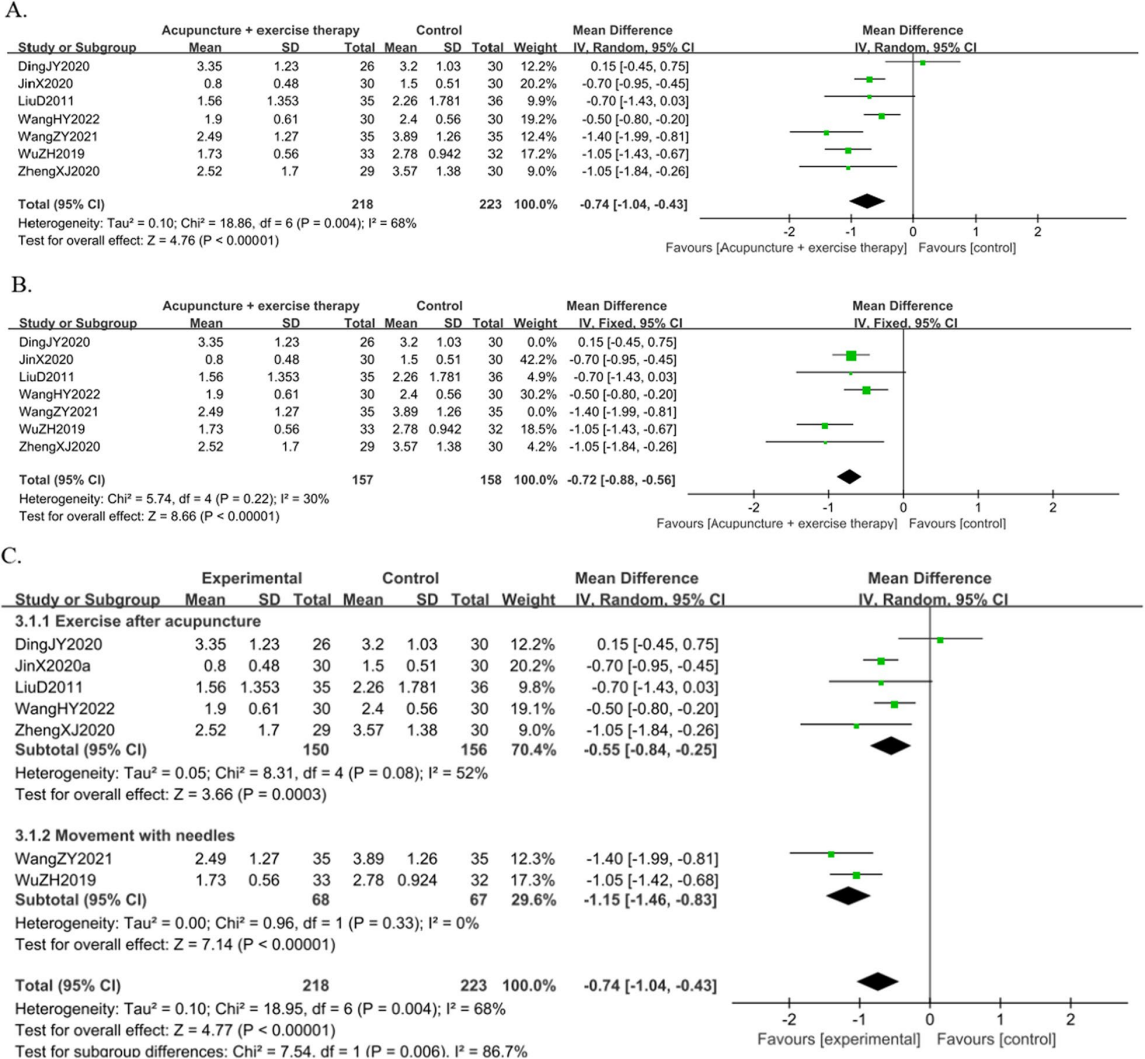
Forest plot of VAS in comparison with combination group versus acupuncture group. (A) All studies. (B) After sensitivity analysis. (C) After subgroup analysis

A total of seven studies [9, 19, 21, 23, 25–27] reported the VAS score in 441 KOA individuals. The results showed that the combined group was better at reducing pain than the acupuncture group [MD =  − 0.74, 95%CI (− 1.04, − 0.43),* I*^*2*^ = 68%, *P* < 0.05] (Fig. 7A). By exploring heterogeneity, we found the risk of bias in two trials [9, 27] was high. After removing the two trials (the duration of treatment in these two trials was not 4 weeks, while the other trials were all 4 weeks), sensitivity analysis showed that the overall effects did not change [MD =  − 0.72, 95%CI (− 0.88, − 0.56), *I*^*2*^ = 30%,* P* = 0.22] (Fig. 7B).

Because of the heterogeneity of the VAS score, a subgroup analysis of active exercise training after acupuncture and active exercise training with needles showed that heterogeneity was reduced in both groups [MD =  − 0.55, 95%CI (− 0.84, − 0.25),* I*^*2*^ = 52%, *P* = 0.08], [MD =  − 1.15, 95%CI (− 1.46, − 0.83), *I*^*2*^ = 0%, *P* = 0.33] (Fig. 7C). The results of the subgroup analysis also suggested that exercise during acupuncture might be more effective than exercise after acupuncture in reducing pain.

#### Result of the WOMAC total score

**Fig. 8:**
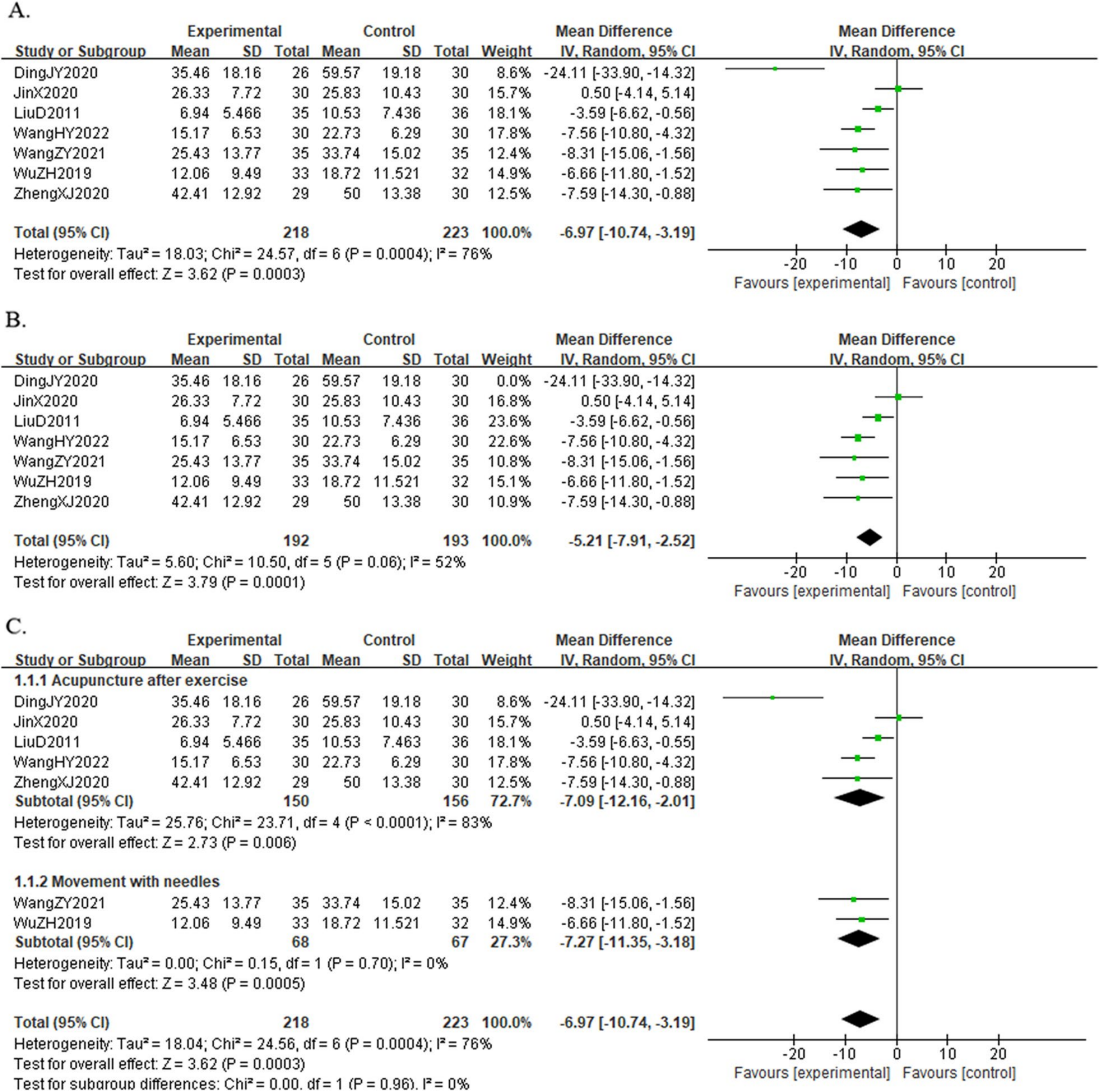
Forest plot of WOMAC total score in comparison with combination group versus acupuncture group. (A) All studies. (B) After sensitivity analysis. (C) After subgroup analysis

A total of seven studies [9, 19, 21, 23, 25–27] reported the WOMAC total score in 441 KOA individuals. The results showed that the combined group was better at relieving knee symptoms and improving knee function than the acupuncture group [MD =  − 6.97, 95%CI (− 10.74, − 3.19), *I*^*2*^ = 76%, *P* < 0.05] (Fig. 8A). By analyzing the sources of WOMAC heterogeneity, we found that after excluding one trial [22] (duration of treatment for 5 weeks, frequency of treatment twice a week), heterogeneity was reduced[MD =  − 5.21, 95%CI (− 7.91, − 2.52), *I*^*2*^ = 52%, *P* = 0.06] (Fig. 8B).

Due to the heterogeneity of the results, we also performed a subgroup analysis based on the time of exercise intervention (active exercise training after acupuncture or active exercise training with needles), which showed that knee function improved in both groups [MD =  − 7.09, 95%CI (− 12.16, − 2.01),* I*^*2*^ = 83%, *P* < 0.05], [MD =  − 7.27, 95%CI (− 11.35, − 3.28), *I*^*2*^ = 0%, *P* = 0.7] (Fig. 8C). The results of the subgroup analysis also suggested that exercise during acupuncture might be more effective in improving knee joint function than exercise after acupuncture.

**Fig. 9:**
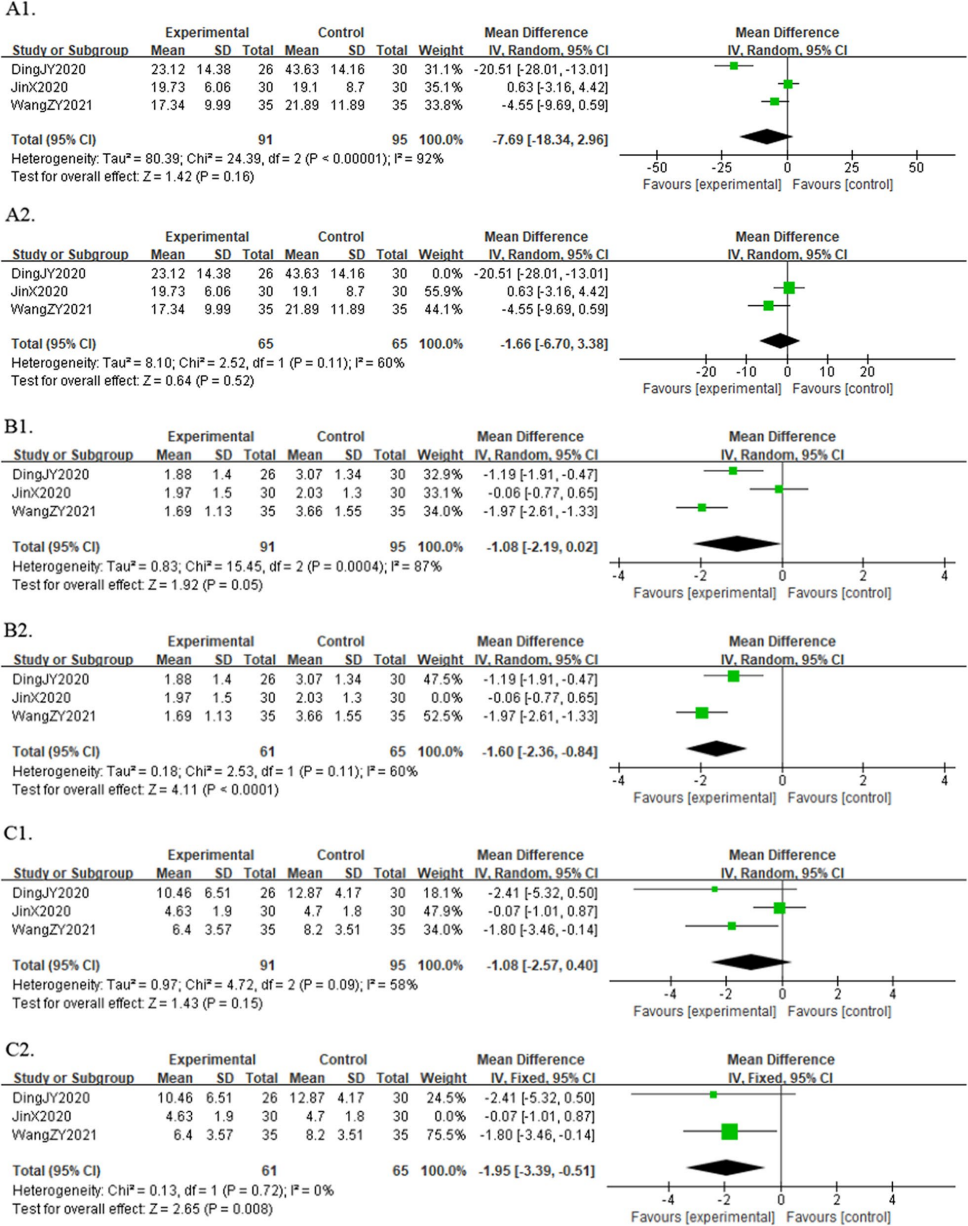
Forest plot of WOMAC-dysfunction (A), WOMAC-stiffness (B), WOMAC-pain (C) in comparison with combination group versus acupuncture group. (1) All studies. (2) After sensitivity analysis

*Result of the WOMAC-dysfunction* A total of three trials [9, 19, 27] reported WOMAC-dysfunction in 186 KOA individuals. The results showed that the dysfunction score of the combined group was significantly lower than that of the acupuncture group, indicating that the combined group could better improve the functional status of KOA patients[MD =  − 7.69, 95%CI (− 18.34, 2.96), *I*^*2*^ = 92%, *P* < 0.05] (Fig. 9A1).

By analyzing the sources of WOMAC-dysfunction heterogeneity, we performed a sensitivity analysis. We found that the heterogeneity decreased after excluding one trial [27] with the duration of treatment for 5 weeks and frequency of treatment twice a week [MD =  − 1.66, 95%CI (− 6.70, 3.38), *I*^*2*^ = 60%, *P* = 0.11] (Fig. 9A2).

*Result of the WOMAC-stiffness* A total of three trials [9, 19, 27] reported WOMAC-stiffness in 186 KOA individuals. The results showed that the combined group was better at relieving knee stiffness than the acupuncture group[MD =  − 1.08, 95%CI (− 2.19, 0.02), *I*^*2*^ = 87%, *P* < 0.05] (Fig. 9B1). By analyzing the sources of WOMAC-stiffness heterogeneity, we found that heterogeneity was reduced after excluding a three-arm study [19] [MD =  − 1.60, 95%CI (− 2.36, − 0.84), *I*^*2*^ = 60%, *P* = 0.11] (Fig. 9B2).

*Result of the WOMAC-pain* A total of three trials [9, 19, 27] reported WOMAC-pain in 186 KOA individuals. The results showed that the combined group was better at relieving pain than the acupuncture group[MD =  − 1.08, 95%CI (− 2.57, 0.40), *I*^*2*^ = 58%, *P* = 0.09] (Fig. 9C1). By analyzing the sources of WOMAC-pain heterogeneity, we found that heterogeneity was reduced after excluding a three-arm study [19] [MD =  − 1.95, 95%CI (− 3.39, − 0.51), *I*^*2*^ = 0%, *P* = 0.72] (Fig. 9C2).

#### Result of the ROM

**Fig. 10:**
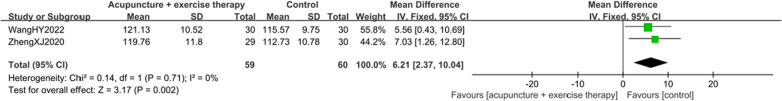
Forest plot of ROM in comparison with combination group versus acupuncture group

A total of two studies [23, 26] reported the ROM in 119 KOA individuals**.** The results showed that the combined group was better at improving joint range of motion than the acupuncture group [MD = 6.25, 95%CI (2.37, 10.04), *I*^*2*^ = 0%, *P* = 0.71] (Fig. 10).

### Combination group versus exercise or western medicine or exercise medicine group

#### Result of the total efficiency rate

**Fig. 11:**
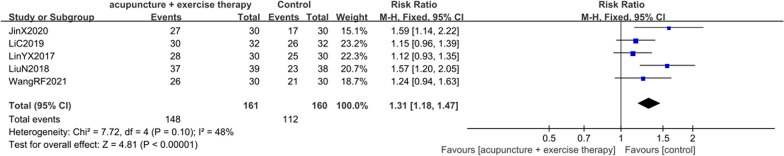
Forest plot of total efficiency rate in comparison with combination group versus exercise or western medicine or exercise medicine group

A total of five studies [19, 20, 22, 24, 28] involving 321 KOA individuals compared the total efficiency rate of acupuncture combined with active exercise training with exercise or western medicine or exercise medicine on KOA. The results demonstrated that combination group was superior to control group in the total efficiency rate [RR = 1.31, 95% CI (1.18, 1.47), *I*^*2*^ = 48%, *P* = 0.10] (Fig. 11).

#### Result of the VAS

**Fig. 12:**
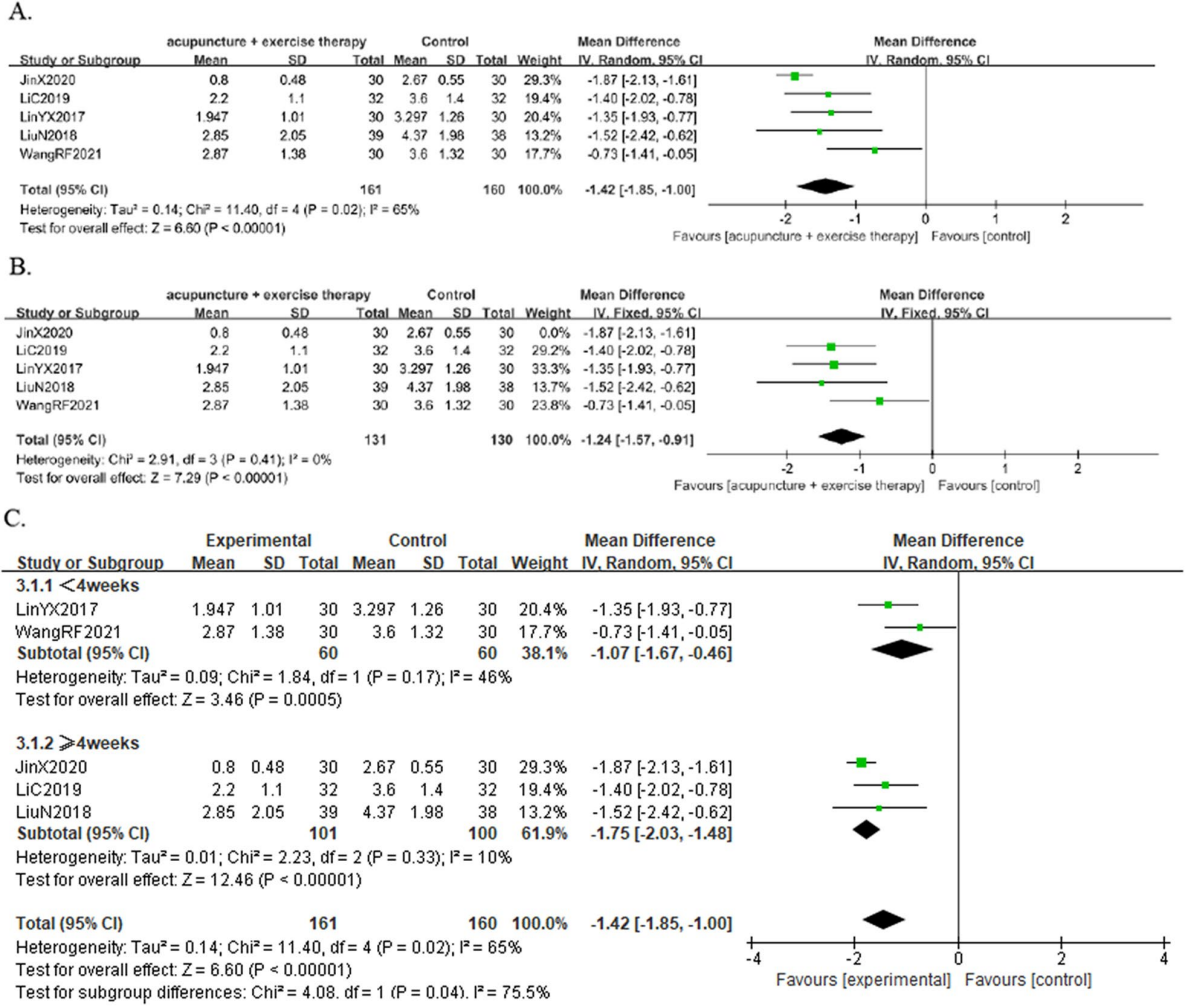
Forest plot of VAS in comparison with combination group versus exercise or western medicine or exercise medicine group. (A) All studies. (B) After sensitivity analysis. (C) After subgroup analysis

A total of five studies [19, 20, 22, 24, 28] reported the VAS in 321 KOA individuals. The results showed that the combined group was better at reducing pain than the control group [MD = 1.42, 95% CI (− 1.85, − 1.00),* I*^*2*^ = 65%, *P* = 0.02] (Fig. 12A). By exploring heterogeneity, we found that the heterogeneity decreased after excluding a three-arm trial [19] with only exercise and no other treatment[MD =  − 1.24, 95%CI (− 1.57, − 0.91), *I*^*2*^ = 0%, *P* = 0.41] (Fig. 12B).

Due to the heterogeneity of the results, we further performed a subgroup analysis of the duration of treatment. The results showed that heterogeneity was reduced in both groups [MD =  − 1.07, 95%CI (− 1.67, − 0.46), *I*^*2*^ = 46%, *P* = 0.17], [MD =  − 1.75, 95%CI (− 2.03, − 1.48), *I*^*2*^ = 10%, *P* = 0.33] (Fig. 12C). The results of the subgroup analysis also indicated that the longer the treatment period, the more pain reduction in KOA individuals.

#### Result of the WOMAC total score

**Fig. 13:**
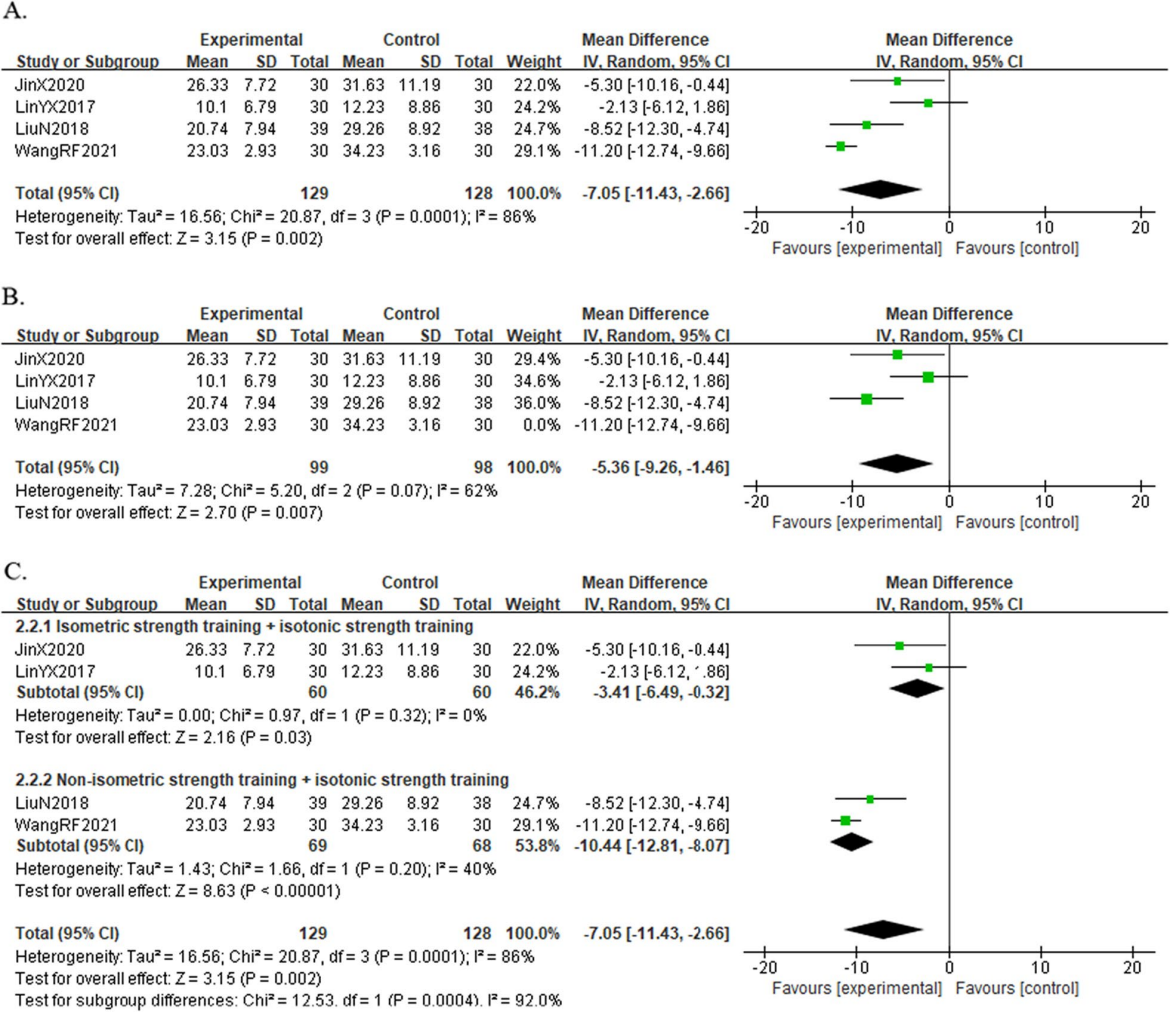
Forest plot of WOMAC total score in comparison with combination group versus exercise or western medicine or exercise medicine group. (A) All studies. (B) After sensitivity analysis. (C) After subgroup analysis

A total of four studies [19, 20, 22, 24] reported the WOMAC total score in 257 KOA individuals. The results showed that the combined group was better at relieving knee symptoms and improving knee function than the control group [MD =  − 7.05, 95%CI (− 11.43, − 2.66), *I*^*2*^ = 86%, *P* < 0.05] (Fig. 13A). By analyzing the sources of WOMAC heterogeneity, we found that after excluding one trial [24] with exercise during acupuncture and treatment duration of 20 days, heterogeneity was reduced [MD =  − 5.36, 95%CI (− 9.26, − 1.46), *I*^*2*^ = 62%, *P* = 0.07] (Fig. 13B).

Due to the heterogeneity of the results, we also performed a subgroup analysis based on the type of exercise (isometric strength training + isotonic strength training or not), which showed that knee function improved in both groups [MD =  − 3.41, 95%CI (− 6.49, − 0.32), *I*^*2*^ = 0%, *P* = 0.32], [MD =  − 10.44, 95%CI (− 12.81, − 8.07), *I*^*2*^ = 40%, *P* = 0.20] (Fig. 13C).

**Fig. 14:**
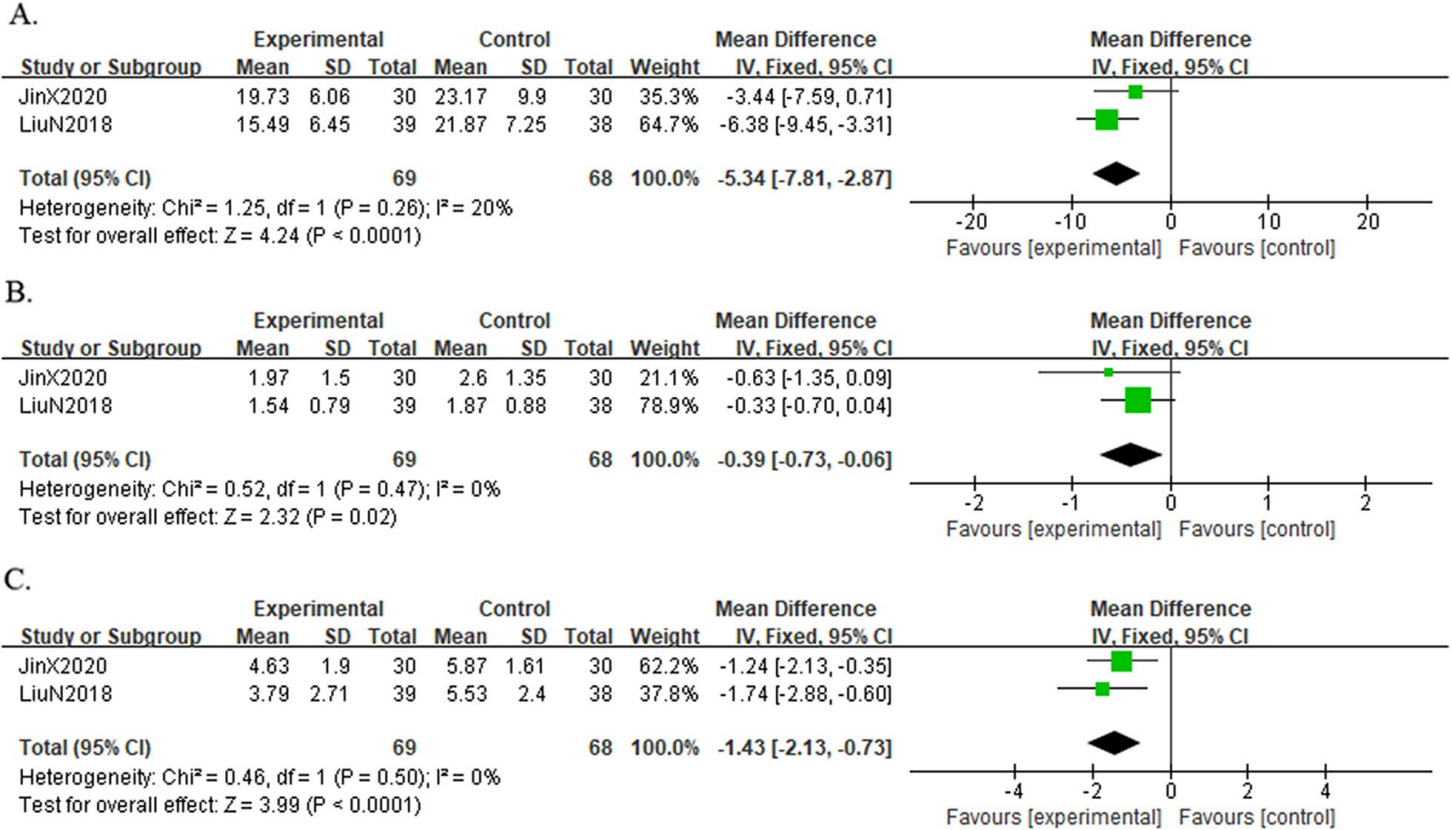
Forest plot of WOMAC-dysfunction (A), WOMAC-stiffness (B), WOMAC-pain (C) in comparison with combination group versus exercise or western medicine or exercise medicine group

*Result of the WOMAC-dysfunction* A total of two trials [19, 22] reported WOMAC-dysfunction in 137 KOA individuals. The results showed that the dysfunction score of the combined group was significantly lower than that of the control group, indicating that the combined group could better improve the functional status of KOA individuals [MD =  − 5.34, 95%CI (− 7.81, − 2.87), *I*^*2*^ = 20%, *P* = 0.26] (Fig. 14A).

*Result of the WOMAC-stiffness* A total of two trials [19, 22] reported WOMAC-stiffness in 137 KOA individuals. The results showed that the combined group was better at relieving knee stiffness than the control group [MD =  − 0.39, 95%CI (− 0.73, − 0.06), *I*^*2*^ = 0%, *P* = 0.47] (Fig. 14B).

*Result of the WOMAC-pain* A total of two trials [19, 22] reported WOMAC-pain in 137 KOA individuals. The results showed that the combined group was better at relieving pain than the control group [MD =  − 1.43, 95%CI (− 2.13, 0.73), *I*^*2*^ = 0%, *P* = 0.50] (Fig. 14C).

## Certainty of the evidence

The results of the GRADE are shown in Additional file 4. The certainty of the evidence of total efficiency rate (combination group versus acupuncture group) was graded as “moderate,” and the rest outcomes were considered as “low” or “very low.” The reasons for downgrading were mainly attributed to the risk of bias of included studies and imprecision and publication bias generated by small sample sizes.

## Discussion

This systematic review and meta-analysis of 11 studies involving 774 KOA individuals aimed at assessing the effectiveness of acupuncture combined with active exercise training on KOA and improvement in knee pain and function. Ultimately, the results of our study indicated that acupuncture combined with active exercise training might be an effective treatment for KOA individuals.

The results of the meta-analysis were generally stable, but the analysis of the WOMAC score required more discussion. The WOMAC was mainly composed of dysfunction, stiffness and pain. In addition to statistical analysis of the WOMAC total score, we also combined the effect size of dysfunction, stiffness and pain, respectively. However, when comparing the combined group with the acupuncture group, we found statistical heterogeneity in WOMAC-dysfunction (*I*^*2*^ = 92%, *P* < 0.05), WOMAC-stiffness (*I*^*2*^ = 87%,* P* < 0.05) and WOMAC-pain (*I*^*2*^ = 58%, *P* = 0.09) after combined effect size, respectively. Therefore, we conducted sensitivity analysis to find the source of heterogeneity, and only the heterogeneity of pain indicators was significantly reduced (*I*^*2*^ = 0%, *P* = 0.72), while the heterogeneity of dysfunction (I^2^ = 60%, P = 0.11) and stiffness (*I*^*2*^ = 60%, *P* = 0.11) decreased after sensitivity analysis, but it was still more than 50%, which was not suitable for subgroup analysis and other analyses due to the limitation of the number of included studies. When the combined group was compared with the non-acupuncture group, there was no statistical heterogeneity in dysfunction (*I*^*2*^ = 20%, *P* = 0.26), stiffness (*I*^*2*^ = 0%, *P* = 0.47) and pain (*I*^2^ = 0%, *P* = 0.50), and the results were stable. Anyhow, almost all results showed that the combination therapy significantly improved functional activity, alleviated joint stiffness and reduced pain in KOA individuals.

At present, the etiology and pathophysiological mechanism of KOA are not clear. And some studies [29–34] believed that the factors leading to the occurrence of KOA included age, obesity, environmental and genetic factors, malnutrition, joint ligament injury, meniscal injury, knee fracture and knee instability. Currently, the main clinical measures for the treatment of KOA include NSAIDs, intra-articular injection, physical therapy, traditional Chinese medicine treatment, exercise therapy and other conservative treatment and surgical treatment [35]. In recent years, complementary and alternative medicine (CAM) has been widely accepted and applied in clinical practice. As complementary alternative therapies, acupuncture and exercise therapy have obvious clinical effects on KOA, and there are no obvious side effects compared with surgical and western drugs, and patients have a high degree of acceptance. According to Chinese medicine, KOA belongs to the category of “paralysis” and “bone paralysis,” which are generally caused by insufficient qi and blood, liver and kidney deficiency and external evil invasion and damage to the knee joint [36]. Commonly used acupuncture tools for clinical knee joint treatment include millineedle, electroacupuncture, floating needle, etc. [37]. Relevant studies have shown that acupuncture can significantly reduce pain, improve dysfunction and improve the quality of life of KOA individuals [15, 38]. The mechanism of action of acupuncture in the treatment of KOA includes: (1) Acupuncture can scavenge free radicals [39], inhibit the expression of osteopontin (OPN), matrix metalloproteinase-3 (MMP-3), transforming growth factor-β1 (TGF-β1) and insulin-like growth factor I (IGF-I) and serum nitric oxidein peripheral blood and synovial fluid of joints, promote the repair of joint cartilage, relieve and improve local inflammatory symptoms of knee joint and play a role in the treatment of KOA [40–42]. (2) KOA individuals are often accompanied by abnormal muscle function and acupuncture can improve early KOA muscle atrophy, significantly increase the expression of Pax7, MyoD, MyoG, MyHC1 and other muscle-generating molecular markers and Wnt/β-catenin pathway-related gene proteins, promote the proliferation and differentiation of skeletal muscle stem cells to achieve the regeneration and repair of damaged skeletal muscle and have a protective effect on early KOA joint chondrocytes and type II collagen metabolism [43, 44]. (3) One study [1] has shown that compared with other acupuncture, electroacupuncture has a better analgesic and anti-inflammatory mechanism for KOA [45, 46], and electroacupuncture on local acupuncture points of the knee joint can not only repair knee cartilage, but also regulate knee microcirculation, increase endogenous opioid levels and significantly reduce plasma cortisol levels [47–49].

At the same time, many clinical guidelines [50–53] recommend exercise therapy as the main measure to prevent and treat KOA. Exercise therapy can not only delay the degeneration of cartilage tissue, improve muscle strength and restore the normal function of musculoskeletal, but also relieve pain, improve joint mobility, protect soft tissues, promote blood circulation, improve their quality of life and achieve the purpose of improving comprehensive curative effect [54–58]. The mechanism of exercise therapy in the treatment of KOA can be summarized as follows: (1) Inhibit the expression of inflammatory factors such as IL-1β, IL-6, IL-8 and TNF-α, thereby reducing the inflammatory response of patients, improving the immune related indexes such as Tim-3 and PD-1 in KOA individuals and inhibiting their autoimmune response [59]. (2) KOA individuals often have knee stress imbalance and lower extremity mechanical axis abnormalities [60]. Exercise therapy can correct the stress imbalance of the lower limb joints of KOA individuals, adjust the state of soft tissue dysfunction around the joints of the lower limbs, reduce the angle of the joint space and improve the biomechanical indexes [61, 62]. (3) The mechanical signals generated by the squeezing effect on the knee joint during active exercise can regulate the expression of TNF-α, MMP-13 and integrin-α1β1, inhibit the apoptosis of chondrocytes and then delay the degeneration of joint cartilage [61, 63].

Both acupuncture and active exercise therapy have good effects on KOA, and relevant studies [64–66] have shown that combining the two treatment modalities shows more excellent results than acupuncture alone or active exercise training alone or only oral western medicine or exercise combined with western medicine.

Our review was conducted by developing strict inclusion/exclusion criteria and controlling for methodological quality. This meta-analysis searched eight electronic databases to provide a comprehensive study. Moreover, all the included studies were of high quality (Jadad ≥ 4), and the conclusions were relatively reliable. In addition, we conducted a group comparison (combined treatment versus acupuncture alone, combined treatment versus non-acupuncture) to more fully illustrate the effectiveness of acupuncture combined active exercise training for pain and dysfunction in KOA individuals. However, there are several potential limitations in our study. Firstly, some of the studies included in our review had methodological flaws. The most common methodological deficit was lack of blinding of participants, therapists and assessors. Secondly, most of the included studies had small sample sizes (*n* < 50). And larger, high-quality studies are needed for further analysis in future. Thirdly, the types of acupuncture in the included studies had different acupuncture points and the number of acupuncture points selected during treatment, as well as certain differences in the mode and intensity of exercise, which might be the reason for the large heterogeneity. Therefore, a more detailed meta-analysis is needed as a next step. Finally, there were differences in follow-up time for the studies included in this review. Most outcomes were measured after treatment, and only six studies reported follow-up outcomes. The effect of follow-up time on the effect of acupuncture combined with active exercise training on KOA needs to be further explored.

## Conclusion

Meta-analysis showed that acupuncture combined with active exercise training had significant efficacy and few side effects in reducing pain, improving knee function, increasing joint range of motion and overall effective rate in KOA individuals, and deserved further promotion. However, due to some shortcomings in this meta-analysis, further trials and analyses are needed to confirm this.

### Supplementary Information

**Additional file 1**: The Preferred Reporting Items for Systematic reviews and Meta-Analysis (PRISMA) 2020 Main Checklist.

**Additional file 2**: Search strategies of electronic databases.

**Additional file 3**: A list of excluded studies by reading full text.

**Additional file 4**: Quality of evidence per outcome from selected studies by the GRADE approach.

## Data Availability

The original contributions presented in the study are included in the article/Supplementary material. Further inquiries can be directed to the corresponding authors.

## Abbreviations

KOA: Knee osteoarthritis
RCTs: Randomized controlled trials
VAS: Visual analogue scale
WOMAC: Western Ontario and Mcmaster Universities Osteoarthritis Index
LKS: Lysholm Knee Scale
ROM: Range of motion
MD: Mean difference
SD: Standard deviation
CI: Confidence interval
CNKI: China national knowledge infrastructure
VIP: Technology periodical database
CBM: China biology medicine
AT: Acupuncture
TSA: Trial sequential analysis
