# Supplementary material for: Efficacy of acupuncture combined with active exercise training in improving pain and function of knee osteoarthritis individuals: a systematic review and meta-analysis
Source: J Orthop Surg Res. 2023 Dec 2;18:921. doi: 10.1186/s13018-023-04403-2 (PMC10693122; doi:10.1186/s13018-023-04403-2)

**Search strategy for PubMed**

#1 Acupuncture [MeSH Terms]

#2(Acupuncture Therapy [Title/Abstract]) OR (Pharmacopuncture [Title/Abstract])

#3 #1 OR #2

#4 Exercise [MeSH Terms]

#5(Exercises [Title/Abstract]) OR (Physical Activity[Title/Abstract]) OR (Physical Activities[Title/Abstract]) OR (Physical Exercise[Title/Abstract]) OR (Physical Exercises[Title/Abstract]) OR (Acute Exercise[Title/Abstract]) OR (Acute Exercises[Title/Abstract]) OR (Isometric Exercises[Title/Abstract]) OR (Isometric Exercise[Title/Abstract]) OR (Aerobic Exercises[Title/Abstract]) OR (Aerobic Exercise[Title/Abstract]) OR (Exercise Trainings[Title/Abstract]) OR (Exercise Training[Title/Abstract]) OR (Rehabilitation[Title/Abstract]) OR (Habilitation[Title/Abstract]) OR (Physical Conditioning, Human[Title/Abstract]) OR (Human Physical Training[Title/Abstract]) OR (Exercise Therapy[Title/Abstract]) OR (Exercise, Remedial[Title/Abstract]) OR (Exercises, Remedial[Title/Abstract]) OR (Exercise Therapies[Title/Abstract]) OR (Rehabilitation Exercise[Title/Abstract]) OR (Rehabilitation Exercises[Title/Abstract]) OR (Resistance Training[Title/Abstract]) OR (Strengthening Programs, Weight-Lifting[Title/Abstract]) OR (Weight Lifting Strengthening Program[Title/Abstract]) OR (Weight Lifting Exercise Program[Title/Abstract]) OR (Weight-Lifting Exercise Programs[Title/Abstract]) OR (Weight Bearing Strengthening Program[Title/Abstract]) OR (Weight-Bearing Strengthening Programs[Title/Abstract]) OR (Weight Bearing Exercise Program[Title/Abstract]) OR (Weight-Bearing Exercise Programs[Title/Abstract]) OR (strength training[Title/Abstract]) OR (muscle training[Title/Abstract]) OR (exercise training[Title/Abstract]) OR (Tai Ji[Title/Abstract]) OR (Yijinjing[Title/Abstract]) OR (Baduanjin[Title/Abstract]) OR (Wuqinxi[Title/Abstract]) OR (Health Qigong[Title/Abstract])

#6 #4 OR #5

#7 Osteoarthritis, Knee[MeSH Terms]

#8(Knee Osteoarthritides[Title/Abstract]) OR (Knee Osteoarthritis[Title/Abstract]) OR (Osteoarthritis of Knee[Title/Abstract]) OR (Osteoarthritis of the Knee[Title/Abstract])

#9 #7 OR #8

#10 #3 AND #6 AND #9

**Search strategy for EMBASE**

#1 ‘Acupuncture ’/exp

#2 ‘Acupuncture Therapy’:ti,ab,kw OR ‘Pharmacopuncture’:ti,ab,kw

#3 #1 OR #2

#4 ‘Exercise’ /exp

#5 ‘Exercises’:ti,ab,kw OR ‘Physical Activity’:ti,ab,kw OR ‘Physical Activities’:ti,ab,kw OR ‘Physical Exercise’:ti,ab,kw OR ‘Physical Exercises’:ti,ab,kw OR ‘Acute Exercise’:ti,ab,kw OR ‘Acute Exercises’:ti,ab,kw OR ‘Isometric Exercises’:ti,ab,kw OR ‘Isometric Exercise’:ti,ab,kw OR ‘Aerobic Exercises’:ti,ab,kw OR ‘Aerobic Exercise’:ti,ab,kw OR ‘Exercise Trainings’:ti,ab,kw OR ‘Exercise Training’:ti,ab,kw OR ‘Rehabilitation’:ti,ab,kw OR ‘Habilitation’:ti,ab,kw OR ‘Physical Conditioning, Human’:ti,ab,kw OR ‘Human Physical Training’:ti,ab,kw OR ‘Exercise Therapy’:ti,ab,kw OR ‘Exercise, Remedial’:ti,ab,kw OR ‘Exercises, Remedial’:ti,ab,kw OR ‘Exercise Therapies’:ti,ab,kw OR ‘Rehabilitation Exercise’:ti,ab,kw OR ‘Rehabilitation Exercises’:ti,ab,kw OR ‘Resistance Training’:ti,ab,kw OR ‘Strengthening Programs, Weight-Lifting’:ti,ab,kw OR ‘Weight Lifting Strengthening Program’:ti,ab,kw OR ‘Weight Lifting Exercise Program’:ti,ab,kw OR‘Weight-Lifting Exercise Programs’:ti,ab,kw OR‘Weight Bearing Strengthening Program’:ti,ab,kw OR‘Weight-Bearing Strengthening Programs’:ti,ab,kw OR‘Weight Bearing Exercise Program’:ti,ab,kw OR‘Weight-Bearing Exercise Programs’:ti,ab,kw OR ‘strength training’:ti,ab,kw OR ‘muscle training’:ti,ab,kw OR ‘exercise training’:ti,ab,kw OR ‘Tai Ji’:ti,ab,kw OR ‘Yijinjing’:ti,ab,kw OR ‘Baduanjin’:ti,ab,kw OR ‘Wuqinxi’:ti,ab,kw OR ‘Health Qigong’:ti,ab,kw

#6 #4 OR #5

#7 ‘Osteoarthritis, Knee’ /exp

#8 ‘Knee Osteoarthritides’:ti,ab,kw OR ‘Knee Osteoarthritis’:ti,ab,kw OR ‘Osteoarthritis of Knee’:ti,ab,kw OR ‘Osteoarthritis of the Knee’:ti,ab,kw

#9 #7 OR #8

#10 #3 AND #6 AND #9

**Search strategy for The Cochrane Library**

#1 MeSH descriptor: [Acupuncture] explode all trees

#2 (Acupuncture Therapy):ti,ab,kw OR (Pharmacopuncture):ti,ab,kw

#3 #1 OR #2

#4 MeSH descriptor: [Exercise] explode all trees

#5 (Exercises):ti,ab,kw OR (Physical Activity):ti,ab,kw OR (Physical Activities):ti,ab,kw OR (Physical Exercise):ti,ab,kw OR (Physical Exercises):ti,ab,kw OR (Acute Exercise):ti,ab,kw OR (Acute Exercises):ti,ab,kw OR (Isometric Exercises):ti,ab,kw OR (Isometric Exercise):ti,ab,kw OR (Aerobic Exercises):ti,ab,kw OR (Aerobic Exercise):ti,ab,kw OR (Exercise Trainings):ti,ab,kw OR (Exercise Training):ti,ab,kw OR (Rehabilitation):ti,ab,kw OR (Habilitation):ti,ab,kw OR (Physical Conditioning, Human):ti,ab,kw OR (Human Physical Training):ti,ab,kw OR (Exercise Therapy):ti,ab,kw OR (Exercise, Remedial):ti,ab,kw OR (Exercises, Remedial):ti,ab,kw OR (Exercise Therapies):ti,ab,kw OR (Rehabilitation Exercise):ti,ab,kw OR (Rehabilitation Exercises):ti,ab,kw OR (Resistance Training):ti,ab,kw OR (Strengthening Programs, Weight-Lifting):ti,ab,kw OR (Weight Lifting Strengthening Program):ti,ab,kw OR (Weight Lifting Exercise Program):ti,ab,kw OR (Weight-Lifting Exercise Programs):ti,ab,kw OR (Weight Bearing Strengthening Program):ti,ab,kw OR (Weight-Bearing Strengthening Programs):ti,ab,kw OR (Weight Bearing Exercise Program):ti,ab,kw OR (Weight-Bearing Exercise Programs):ti,ab,kw OR (strength training):ti,ab,kw OR (muscle training):ti,ab,kw OR (exercise training):ti,ab,kw OR (Tai Ji):ti,ab,kw OR (Yijinjing):ti,ab,kw OR (Baduanjin):ti,ab,kw OR (Wuqinxi):ti,ab,kw OR (Health Qigong):ti,ab,kw

#6 #4 OR #5

#7 MeSH descriptor: [Osteoarthritis, Knee] explode all trees

#8 (Knee Osteoarthritides):ti,ab,kw OR (Knee Osteoarthritis):ti,ab,kw OR (Osteoarthritis of Knee):ti,ab,kw OR (Osteoarthritis of the Knee):ti,ab,kw

#9 #7 OR #8

#10 #3 AND #6 AND #9

**Search strategy for Web of science**

#1 TS=(Acupuncture OR Acupuncture Therapy OR Pharmacopuncture)

#2 TS=(Exercise OR Exercises OR Physical Activity OR Physical Activities OR Physical Exercise OR Physical Exercises OR Acute Exercise OR Acute Exercises OR Isometric Exercises OR Isometric Exercise OR Aerobic Exercises OR Aerobic Exercise OR Exercise Trainings OR Exercise Training OR Rehabilitation OR Habilitation OR Physical Conditioning, Human OR Human Physical Training OR Exercise Therapy OR Exercise, Remedial OR Exercises, Remedial OR Exercise Therapies OR Rehabilitation Exercise OR Rehabilitation Exercises OR Resistance Training OR Strengthening Programs, Weight-Lifting OR Weight Lifting Strengthening Program OR Weight Lifting Exercise Program OR Weight-Lifting Exercise Programs OR Weight Bearing Strengthening Program OR Weight-Bearing Strengthening Programs OR Weight Bearing Exercise Program OR Weight-Bearing Exercise Programs OR strength training OR muscle training OR exercise training OR Taijiquan OR Yijinjing OR Baduanjin OR Wuqinxi OR Health Qigong)

#3 TS=(Osteoarthritis,Knee OR Knee Osteoarthritides OR Knee Osteoarthritis OR Osteoarthritis of Knee OR Osteoarthritis of the Knee OR knee osteoarthritis)

#4 (#1 AND #2 AND #3)

**Search strategy for CNKI**

（（主题：针刺（精确））OR（主题：针灸（精确））AND （（主题：运动疗法（精确））OR（主题：康复（精确））OR（主题：抗阻训练（精确））OR（主题：肌力训练（精确））OR（主题：功能锻炼（精确））OR（主题：体能锻炼（精确））OR（主题：太极拳（精确））OR（主题：八段锦（精确））OR（主题：易筋经（精确））OR（主题：健身气功（精确）））AND （（主题：膝骨关节炎（精确））OR（主题：膝关节骨性关节炎（精确））OR（主题：膝骨性关节炎（精确）））

**Search strategy for WangFang**

( 主题:(针刺) or 题名或关键词:(针灸)) and ( 主题:(运动疗法) or 题名或关键词:(运动 or 康复 or 抗阻训练 or 训练 or 肌力训练 or 功能锻炼 or 体能锻炼 or 太极拳 or 八段锦 or 易筋经 or 五禽戏 or 健身气功) ) and ( 主题:(膝骨关节炎) or 题名或关键词:(膝关节骨性关节炎 or 膝骨性关节炎) )

**Search strategy for VIP**

(题名或关键词=针刺 OR 针灸) AND (题名或关键词=运动疗法OR运动 OR 康复 OR 抗阻训练 OR 训练 OR 肌力训练 OR 功能锻炼 OR 体能锻炼 OR 太极拳 OR 八段锦 OR 易筋经 OR 五禽戏 OR 健身气功) AND (题名或关键词=膝骨关节炎 OR 膝关节骨性关节炎 OR 膝骨性关节炎)

**Search strategy for CBM**


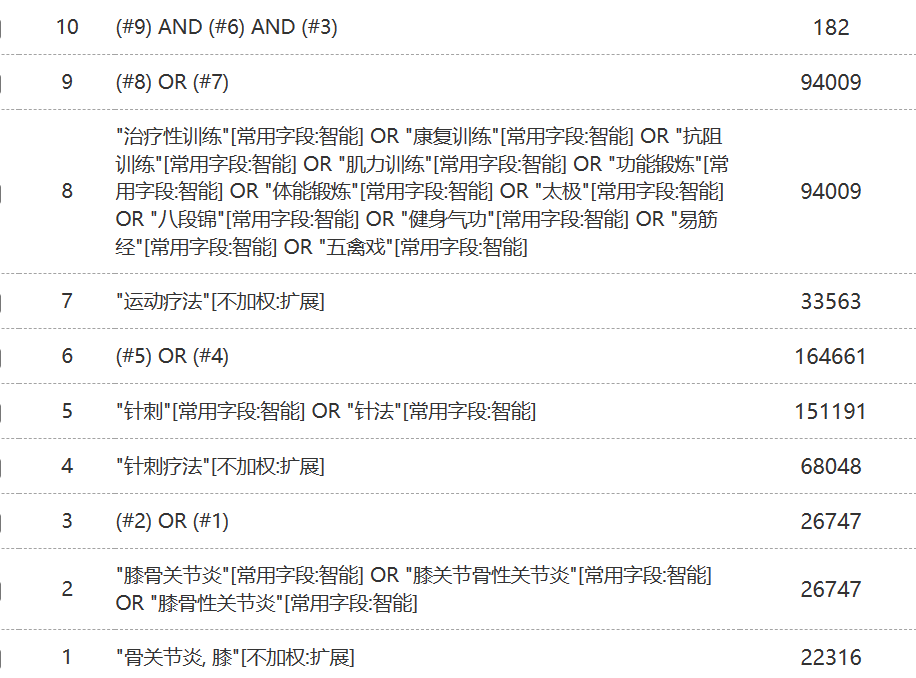

Supplement: Supplementary file 2 — Additional file 2: Search strategies of electronic databases. [file 13018_2023_4403_MOESM2_ESM.docx]
