# Supplementary material for: Efficacy of acupuncture combined with active exercise training in improving pain and function of knee osteoarthritis individuals: a systematic review and meta-analysis
Source: J Orthop Surg Res. 2023 Dec 2;18:921. doi: 10.1186/s13018-023-04403-2 (PMC10693122; doi:10.1186/s13018-023-04403-2)
Supplement: Supplementary file 3 — Additional file 3: A list of excluded studies by reading full text. [file 13018_2023_4403_MOESM3_ESM.docx]

**A list of excluded studies by reading full text.**

**Not RCT(n=22):**

1. Hou Meijin. Effects of electricity on functional activity and ladder gait in patients with knee osteoarthritis[D].Fujian University of Chinese Medicine,2017.

2. ZHENG Shuang,LIU Peng,MAO Xudong,et al. Efficacy of acupuncture combined with strength training in the treatment of knee osteoarthritis[J].World Latest Medical Information Digest,2016,16(28):130-131. DOI:10.3969/j.issn.1671-3141.2016.28.097

3. Li Tianjiao, Li Xiang. Acupuncture combined with isokinetic muscle strength training of 30 cases of treatment of knee osteoarthritis [J]. Journal of fujian TCM, 2016,47 (01) : 2-3. DOI: 10.13260 / j.carol carroll nki JFJTCM. 011091.

4. Chen Shiping. Effect of acupuncture therapy combined with rehabilitation guidance on patients with knee osteoarthritis [J]. Journal of Contemporary Medicine,2016,14(10):22-23.

5. Cao Shuli, Jin Peng. 23 cases of knee osteoarthritis treated by acupuncture exercise therapy [J]. Chinese Journal of Acupuncture and Moxibustion,2014,34(09):906.DOI:10.13703/J.0255-2930.2014.09.028.

6. Deng Qilong, Wu Huan, Sun Guojie et al. Joint acupuncture combined with exercise therapy for knee osteoarthritis [J]. World of Chinese Medicine,2013,8(01):76-77. DOI:10.3969/j.issn.1673-7202.2013.01.031.

7. Niu Xiaojun. Acupuncture combined with rehabilitation training for the treatment of knee osteoarthritis: 78 cases[J].Chinese Journal of Ethnic and Folk Medicine,2010,19(17):187. DOI:10.3969/j.issn.1007-8517.2010.17.144.

8. LIU Qiang, WEI Min, LI Min, et al. Acupuncture combined with exercise therapy for the treatment of knee osteoarthritis: 80 cases[J].Guangxi Journal of Traditional Chinese Medicine,2009,32(04):29-30. DOI:10.3969/j.issn.1003-0719.2009.04.015.

9. ZHAO Liqing,HUANG Yanxing,TENG Weiran. Evaluation of the efficacy of acupuncture combined with rehabilitation guidance in the treatment of knee osteoarthritis[J].Chinese Journal of Clinical Rehabilitation,2005(31):149-151. DOI:10.3321/j.issn:1673-8225.2005.31.061.

10. Qu Xiaodong, Zhou Jingjie, Zhai Hongwei, et al. Efficacy of exercise acupuncture and osteopathic therapy in the treatment of traumatic knee arthritis[J].Chinese Journal of Bone Injury,2019,32(06):493-497. DOI:10.3969/j.issn.1003-0034.2019.06.002.

11. REN Yun,ZHANG Jiao,WEI Jinyang,ZHANG Yifang. Clinical effect of small needle knife combined with exercise therapy in the treatment of knee osteoarthritis[J].China Primary Medicine,2015,0(24):3770-377312. DOI:10.3760/cma.j.issn.1008-6706.2015.24.030.

12. Shuai Kuang, Jin Shuwen, Li Yunfang, et al. Clinical observation of acupuncture combined with functional exercise in the treatment of knee arthritis[J]. World Latest Medical Information Abstracts, 2020, 20(71): 190-191. DOI:10.3969/j.issn.1671-3141.2020.71.090.

13. LIN Jianjun, CHEN Qingyun, HUANG Weixu, et al. Clinical effective rate analysis of electroacupuncture in the treatment of stasis blocking knee osteoarthritis[J]. Xinjiang Journal of Traditional Chinese Medicine,2020,38(6):26-28.

14. Yin Yi, Zhao Yan. A case-control study of synergistic effect of electroacupuncture after arthroscopic cleansing of knee osteoarthritis[J]. Chinese Journal of Bone Injury,2014,27(4):287-290. DOI:10.3969/j.issn.1003-0034.2014.04.006.

15. JI Chunnan, LIU Jinjie. Efficacy of acupuncture combined with exercise in the treatment of knee osteoarthritis[J]. China Medical Engineering,2010,18(1):149,151.

16. CAO Hongbo,WANG Simin,GUI Gaofei. Clinical efficacy analysis of electroacupuncture foot with less yang meridian point combined with functional training in the treatment of knee osteoarthritis[J].China Rehabilitation,2020,35(12):645-649. DOI:10.3870/zgkf.2020.12.007.

17. CHENG Z, WEN Jianmin, LIN Xinxiao, et al. Clinical observation of knee arthroscopy combined with electroelectroacupuncture in the treatment of knee osteoarthritis[J]. International Journal of Chinese Materia Medica,2009,31(4):348,353. DOI:10.3760/cma.j.issn.1673-4246.2009.04.036.

18. WANG Xiurong, ZHENG Qikai, GAN Aifang. Auriculo-point seed burial combined with exercise training in the treatment of yang deficiency and cold coagulation knee osteoarthritis: 30 cases[J].Fujian Journal of Traditional Chinese Medicine,2018,49(02):77-78.DOI:10.13260/j.cnki.jfjtcm.011607.

19. CHEN Xiangfang, QIAO Bo, SHANG Yanhui, et al. Clinical study of "tendon stabbing method" in the treatment of knee osteoarthritis[J]. Clinical Journal of Acupuncture,2022,38(7):43-47. DOI:10.19917/j.cnki.1005-0779.022132.

20. LI Yongjin,SHA Jin,LIU Jun. Efficacy of integrated traditional Chinese and Western medicine in the treatment of knee osteoarthritis in 88 cases[J]. Chinese Medical Guide,2013(30):180-181. DOI:10.3969/j.issn.1671-8194.2013.30.140.

21. Li Zhuo. Clinical observation of acupuncture, tuina plus rehabilitation functional exercise in the treatment of postoperative pain and dysfunction of knee osteoarthropathy in 120 cases[J]. Hebei Journal of Traditional Chinese Medicine,2010,32(2):234-235. DOI:10.3969/j.issn.1002-2619.2010.02.046.

22. Zhang Bo. Retrospective analysis of the clinical efficacy of different treatments for 90 cases of knee osteoarthritis[D]. Shaanxi University of Traditional Chinese Medicine,2016. DOI:10.7666/d.D01059988.

**Unavailable data(n=13):**

1.Pang JCY, Fu ASN, Lam SKH, Peng B, Fu ACL. Ultrasound-guided dry needling versus traditional dry needling for patients with knee osteoarthritis: A double-blind randomized controlled trial. PLoS One. 2022 Sep 30;17(9):e0274990. doi: 10.1371/journal.pone.0274990.

2.LI Zhengxiang. Acupuncture and functional exercise were the main treatment for knee osteoarthritis: 124 cases[J].Modern Rehabilitation,2000(12):891. DOI:10.3321/j.issn:1673-8225.2000.12.119.

3. XU W, SUN D, ZHANG D T, et al. Observations on the Efficacy of Interactive Thumbtack Needle Embedding plus Rehabilitation Instruction in Treating Knee Osteoarthritis[J]. Shanghai journal of acupuncture and moxibustion [shang hai zhen jiu za zhi], 2016, 35(8): 997‐1000.

4.HE J G, FAN W J, JIN S S, et al. Clinical efficacy of functional rehabilitation training combined with acupuncture and moxibustion therapy in treating early-mid knee osteoarthritis and its effect on patients' knee function and quality of life[J]. Asia-Pacific Journal of Clinical Oncology, 2022, 18: 88.

5. Luo X, Hou XS, Tian ZY, Meng X, Li SM, Bai P. [Randomized controlled clinical trial of acupuncture treatment for knee osteoarthritis in the early stage]. Zhen Ci Yan Jiu. 2019 Mar 25;44(3):211-5. Chinese. doi: 10.13702/j.1000-0607.180677.

6.IRCT20181017041371N. Magnet therapy ,acupuncture and isometric exercise in treatment knee osteoarthritis [J]. https://trialsearchwhoint/Trial2aspx?TrialID=IRCT20181017041371N1, 2019.

7. CHICTR. Effect of comprehensive rehabilitation therapy on muscle strength, EMG, muscle status, proprioception and balance in postoperative patients with knee osteoarthritis [J]. https://trialsearchwhoint/Trial2aspx?TrialID=ChiCTR1800016778, 2018.

8. JIANG Zonglun,XU Qinghua,ZHANG Yingchun. Clinical study of acupuncture combined with modern rehabilitation methods in the treatment of knee osteoarthritis[J]. Liaoning Journal of Traditional Chinese Medicine,2018,45(2):381-383. DOI:10.13192/j.issn.1000-1719.2018.02.053.

9. ALLAM A, NEGM A, ALASHKAR D. Clinical and radiologic assessment of local therapy in management of primary osteoarthritis of the knee (a prospective study)[J]. Annals of the Rheumatic Diseases, 2014, 73.

10. SUN K, BAO X M, SONG Y C, et al. [Clinical study on the treatment of knee osteoarthritis by acupuncture plus manipulative regulation of knee muscle][J]. Zhongguo Gu Shang, 2010, 23(12): 895-8.

11.SONI A, MUDGE N, JOSHI A, et al. Severe knee osteoarthritis: A study of combined acupuncture and physiotherapy vs home exercise advice in patients awaiting total knee arthroplasty [J]. Rheumatology, 2010, 49: i79-i80.

12.ACTRN. Effectiveness of physiotherapy resources in knee osteoarthritis: a randomized controlled trial with therapeutic exercise, ear acupuncture and transcutaneous electrical nerve stimulation[J]. https://trialsearchwhoint/Trial2aspx?TrialID=ACTRN12610000232033, 2010.

13. NCT. Effectiveness of Acupuncture as an Adjunct to Rehabilitation After Knee Arthroplasty[J]. https://clinicaltrialsgov/show/NCT00935155, 2009.

**Incorrect outcome measures(n=73):**

1. GAO Yuanhang, CHAI Yi, CUI Shuguo, etc. Study on the efficacy of acupuncture "elbow suture" combined with exercise therapy in the treatment of early knee osteoarthritis[J].Journal of Hebei Journal of Traditional Chinese Medicine,2023,38(01):36-38+60.DOI:10.16370/j.cnki.13-1214/r.2023.01.010.

2. Chen Sheng'e. Clinical effect of needle interactive needle insertion combined with rehabilitation therapy in the treatment of knee osteoarthritis[J].Journal of Sino-Foreign Medical Research,2023,21(06):140-143.DOI:10.14033/j.cnki.cfmr.2023.06.035.

3. XIE Lei, XING Yongsheng. Efficacy of acupuncture combined with celecoxib and exercise therapy in the treatment of knee osteoarthritis[J].Shanghai Medical Journal,2022,43(21):42-45+76. DOI:10.3969/j.issn.1006-1533.2022.21.013.

4. Wu Mingde. Evaluation of the efficacy of liver and kidney acupuncture combined with knee rehabilitation exercise training in the treatment of knee osteoarthritis[J].Journal of Chronic Diseases,2021,22(01):152-153+156.DOI:10.16440/j.cnki.1674-8166.2021.01.056.

5. WANG Zhentao, WANG Yinping, XU Yanqin, et al. Effect of acupuncture combined with exercise therapy on joint function and lower limb microcirculation in elderly patients with knee osteoarthritis[J].Chinese Journal of Gerontology,2021,41(04):818-821. DOI:10.3969/j.issn.1005-9202.2021.04.046

6. WANG Mingjie, WANG Yueliang, ZHOU Xuelong, et al. Clinical study of Yao Yi oil acupuncture exercise acupuncture in the treatment of knee arthritis with damp heat obstruction[J].Chinese Journal of Traditional Chinese Medicine,2021,36(08):5087-5091.

7. CHEN Jiaoying. Randomized controlled trial of qihuang needle therapy in the treatment of knee osteoarthritis[D].Guangzhou University of Chinese Medicine, 2022. DOI:10.27044/d.cnki.ggzzu.

2021.000708.

8. Ye Miaofang. Clinical study of acupuncture combined with conventional therapy in the treatment of knee osteoarthritis[J].New Chinese Medicine,2020,52(03):154-157.DOI:10.13457/j.cnki.jncm.

2020.03.046.

9. Li Yongfeng. Acupuncture combined with sodium hyaluronate intraarticular injection plus resistance function exercise in the treatment of knee osteoarthritis in 60 cases[J].Primary Medicine Forum,2018,22(11):1466-1467.DOI:10.19435/j.1672-1721.2018.11.016.

10. Li Xueying. Comparative study of different exercise methods and acupuncture combination on knee osteoarthritis[J].Contemporary Sports Science and Technology,2018,8(36):14-16+18.

DOI:10.16655/j.cnki.2095-2813.2018.36.014.

11. CHEN Li,LAI Xiaojun. Analysis of the effect of small needle knife holistic release surgery, acupuncture therapy combined with exercise therapy in the treatment of knee osteoarthritis[J].Journal of Contemporary Medicine,2018,16(24):197-198.

DOI:10.3969/j.issn.2095-7629.2018.24.143.

12. ZHU Zhi,LI Enhui,ZHU Qingguang. Clinical observation of tai chi exercise combined with acupuncture in the intervention of knee osteoarthritis[J].Shanghai Journal of Traditional Chinese Medicine,2017,51(03):54-56.DOI:10.16305/j.1007-1334.2017.03.017.

13. WANG Haihua,LIU Aiguo,WANG Xin. Effect of acupuncture exercise therapy on efficacy and joint function in patients with knee osteoarthritis[J].World Journal of Traditional Chinese Medicine,2017,12(07):1648-1651. DOI:10.3969/j.issn.1673-7202.2017.07.043.

14. Cui Jie. Acupuncture combined with sodium hyaluronate intraarticular injection plus resistance function exercise in the treatment of knee osteoarthritis: 60 cases[J].Journal of Traditional Chinese Medicine Research,2017,30(01):63-66. DOI:10.3969/j.issn.1001-6910.2017.01.30.

15. CAI Yu, TANG Weidong, CHEN Hong, et al. Clinical study of acupuncture exercise therapy in the treatment of knee osteoarthritis[J].Guangming Traditional Chinese Medicine,2017,32(09):1306-1308. DOI:10.3969/j.issn.1003-8914.2017.09.038.

16. XU Wei, SUN Dan, ZHANG Datong. Shanghai Journal of Acupuncture,2016,35(08):997-1000.DOI:10.13460/j.issn.1005-0957.2016.08.0997.

17. Xu Wei. Clinical efficacy of interactive needle insertion in the treatment of knee osteoarthritis[D].Zhejiang University of Traditional Chinese Medicine,2016.

18. HE Caihui,LIANG Weili. Effects of millifire acupuncture combined with rehabilitation training on WOMAC score and VAS score in patients with early and middle knee osteoarthritis[J].Emergency Journal of Traditional Chinese Medicine,2015,24(12):2228-2230. DOI:10.3969/j.issn.1004-745X.2015.12.057

19. Wang Xiuhua. Effect of acupuncture on functional recovery after total knee resurfacing surgery[J].Liaoning Journal of Traditional Chinese Medicine,2014,41(08):1653-1654.DOI:10.13192/j.issn.1000-1719.2014.08.040.

20. Zhong Rongfang, Zha Heping, Fan Zhiyong, et al. Functional exercise combined with acupuncture in the treatment of knee osteoarthritis: 60 cases[J].Journal of External Treatment of Traditional Chinese Medicine,2013,22(04):8-9. DOI:10.3969/j.issn.1006-978X.2013.04.003.

21. Shi Zhongya, Hu Fenqiang, Chen Yong. Efficacy of short puncture combined with functional training in the treatment of knee osteoarthritis[J].Shanghai Journal of Acupuncture,2012,31(11):826-828. DOI:10.3969/j.issn.1005-0957.2012.11.826.

22. LUO Kaimin, HOU Zhi, YANG Lin, et al. Efficacy of acupuncture combined with strength training in the treatment of knee osteoarthritis[J].Tianjin Journal of Traditional Chinese Medicine,2012,29(01):45-47.

23. Deng Qilong, Wu Huan, Ma Chaoyang, et al. Joint corresponding acupuncture combined with sitting-standing test in the treatment of knee osteoarthritis[J].Chinese Rehabilitation,2012,27(05):370-371. DOI:10.3870/zgkf.2012.05.021.

24. Qi Linjing. Clinical study of shallow exercise therapy in the treatment of knee osteoarthritis[D].Changchun University of Chinese Medicine,2012.

25. ZHAO Yuguang, LUO Shuangxi. Clinical observation of acupuncture combined with exercise therapy in the treatment of knee osteoarthritis[J].China Medical Herald,2008(11):103-104. DOI:10.3969/j.issn.1673-7210.2008.11.065.

26. Marseille. Clinical observation of giant thorn combined with functional exercise in the treatment of knee osteoarthritis[D].Fujian College of Traditional Chinese Medicine,2009.

27. Gu Xiaomei, Xu Xiaomei, Sun Zhicheng. Efficacy of acupuncture combined with strength training in the treatment of senile knee osteoarthritis[J].Practical Geriatrics,2008,22(06):467-469.

28. Jia Jie, Mao Guanglan, Hu Shaohui, et al. Intervention effect of acupuncture combined with functional training on knee osteoarthritis in the elderly[J].Chinese Journal of Clinical Rehabilitation,2005(10):18-19. DOI:10.3321/j.issn:1673-8225.2005.10.008.

29. HU Yuanshui, ZHANG Zhe, HE Min. Observation of the efficacy of acupuncture combined with rehabilitation training in the treatment of knee osteoarthritis[J].Shaanxi Journal of Traditional Chinese Medicine,2015,36(10):1418-1419. DOI:10.3969/j.issn.1000-7369.2015.10.083.

30. Xu Hongtan. Effect of thermal conductive internal heat acupuncture combined with exercise therapy in the treatment of knee osteoarthritis[J]. Chinese Journal of Disability Medicine,2021,29(16):40-41. DOI:10.13214/j.cnki.cjotadm.2021.16.023.

31. Luo Guize. Clinical discussion on the treatment of knee osteoarthritis by acupuncture of contralateral ulnar acupoint combined with knee functional training[J]. Journal of Systems Medicine,2019,4(6):24-25,47. DOI:10.19368/j.cnki.2096-1782.2019.06.024.

32. HU Weimin, LU Wei, CAI Yijia, et al. Clinical study of acupuncture combined with sequential pile guidance training in the treatment of knee osteoarthritis in the elderly[J]. Henan Journal of Traditional Chinese Medicine,2019,39(6):927-931. DOI:10.16367/j.issn.1003-5028.2019.06.0230.

33. ZHOU Kang. Application of acupuncture combined with proprioceptive training in rehabilitation after total knee replacement[D].Hubei University of Chinese Medicine,2018.

34. CUI Zhegang, SHI Hongliang, LI Xiaojing, et al. Efficacy of acupuncture combined with exercise therapy in the treatment of knee osteoarthritis[J]. China Health and Nutrition,2018,28(32):282-283. DOI:10.3969/j.issn.1004-7484.2018.32.430.

35. Jin Xiaoping. Effect of acupuncture combined with rehabilitation training in the treatment of knee osteoarthritis[J]. Everyone Health,2017,11(10):46-47.

36. Zhang Huiming. Clinical study of acupuncture combined with muscle rehabilitation training in the treatment of knee osteoarthritis[C]. Proceedings of the 14th National Conference of Exercise Therapy of Chinese Association of Rehabilitation Medicine. 2016:1-1.

37. When Hua. Acupuncture treatment and rehabilitation training for knee osteoarthritis[J]. Medical Aesthetics and Cosmetology,2014(7):466-466.

38. Zhuo Junhong. Clinical study of exercise acupuncture in the treatment of degenerative knee arthritis[D]. Guangdong:Guangzhou University of Chinese Medicine,2012.

39. Liu Di. Clinical study of acupuncture combined with isometric exercise in the treatment of knee osteoarthritis in the post-tremic plate area[D]. Sichuan:Chengdu University of Traditional Chinese Medicine,2011.

40. DING Jie, WANG Zhibin, CHEN Gen, et al. Efficacy of electroacupuncture stimulation in patients with knee osteoarthritis[J]. Chinese Journal of Geriatric Health Medicine,2022,20(6):67-70. DOI:10.3969/j.issn.1672-2671.2022.06.015.

41. LI Jinqiu, ZHONG Shangcong, YANG Dehui. Clinical observation of electroacupuncture combined with isokinetic strength training in the treatment of early knee osteoarthritis in the elderly[J]. China Medical Sciences,2021,11(9):15-18,22. DOI:10.3969/j.issn.2095-0616.2021.09.005.

42. HUANG Lei, XU Zhiguo. Efficacy of electroacupuncture combined with functional exercise in the treatment of knee osteoarthritis[J].Journal of Guangzhou University of Chinese Medicine,2020,37(01):78-82.DOI:10.13359/j.cnki.gzxbtcm.2020.01.015.

43. Dai Weili, Guan Mingkun. Effect of abdominal needle combined with rehabilitation training on knee pain and motor function in patients with knee arthritis[J]. New Chinese Medicine,2020,52(2):117-120. DOI:10.13457/j.cnki.jncm.2020.02.034.

44. Teng Jinyan, Zha Huarong, Hu Yi. Effect of electricity on arthroscopic rehabilitation of knee osteoarthritis[J]. Chinese Journal of Orthopedics and Traumatology,2018,26(6):23-26.

45. XIONG Deqi, HUANG Binyang, LIU Xiaorui, et al. Clinical observation of muscle strength improvement of core muscle group combined with electricity based on splenic system in patients with knee osteoarthritis[J]. Journal of External Treatment of Traditional Chinese Medicine,2016,25(4):3-5. DOI:10.3969/j.issn.1006-978X.2016.04.001.

46. ZHU Xiaoju, WANG Xiaojie. Application effect of electroacupuncture combined with exercise intervention in elderly people with knee arthritis in cold land[J]. Clinical Journal of Acupuncture,2015(12):7-10.

47. ZHANG Lingling,WANG Gang,DONG Baoqiang. Clinical efficacy of acupuncture combined with meridional stretching in the treatment of knee osteoarthritis[J]. Journal of Liaoning University of Chinese Medicine,2014,16(8):143-145. DOI:10.13194/j.issn.1673-842x.2014.08.047.

48. SHI Suhua, LI Zhigang, QIN Lina, et al. Application of electroacupuncture combined with isokinetic strength training in patients with knee osteoarthritis[J]. World Journal of Integrated Western Medicine,2014,9(11):1234-1236. DOI:10.13935/j.cnki.sjzx.141130.

49. LIANG Jianling, LI Shaomian, BEI Yun. Clinical observation of electroacupuncture combined with exercise therapy in the treatment of knee osteoarthritis[J]. China Medical Herald,2011,8(26):106-108. DOI:10.3969/j.issn.1673-7210.2011.26.046.

50.PENAGOS-MARTíNEZ A A, PATIñO-PALMA B E, RODRíGUEZ-PUERTO O L. Acupuncture and therapeutic exercise in women over 50 with osteoarthritis of the knee. Randomized clinical trial [J]. Revista Internacional de Acupuntura, 2021, 15(1): 15-23.DOI:10.1016/j.acu.2021.02.001

51. Lam WC, Au KY, Qin Z, Wu FM, Chong CO, Jiang F, He Y, Ng BFL, Yeung WF, Lao L, Chen H. Superficial Needling Acupuncture vs Sham Acupuncture for Knee Osteoarthritis: A Randomized Controlled Trial. Am J Med. 2021 Oct;134(10):1286-1294.e2. doi: 10.1016/j.amjmed.2021.05.002. Epub 2021 Jun 11.

52. Dunning J, Butts R, Young I, Mourad F, Galante V, Bliton P, Tanner M, Fernández-de-Las-Peñas C. Periosteal Electrical Dry Needling as an Adjunct to Exercise and Manual Therapy for Knee Osteoarthritis: A Multicenter Randomized Clinical Trial. Clin J Pain. 2018 Dec;34(12):1149-1158. doi: 10.1097/AJP.0000000000000634.

53. Au KY, Chen H, Lam WC, Chong CO, Lau A, Vardhanabhuti V, Mak KC, Jiang F, Lam WY, Wu FM, Chan HN, Ng YW, Ng BF, Ziea ET, Lao L. Sinew acupuncture for knee osteoarthritis: study protocol for a randomized sham-controlled trial. BMC Complement Altern Med. 2018 Apr 23;18(1):133. doi: 10.1186/s12906-018-2195-8.

54.Chen LX, Mao JJ, Fernandes S, Galantino ML, Guo W, Lariccia P, Teal VL, Bowman MA, Schumacher HR, Farrar JT. Integrating acupuncture with exercise-based physical therapy for knee osteoarthritis: a randomized controlled trial. J Clin Rheumatol. 2013 Sep;19(6):308-16. doi: 10.1097/RHU.0b013e3182a21848.

55. Chen G, Gu RX, Xu DD. [The application of electroacupuncture to postoperative rehabilitation of total knee replacement]. Zhongguo Zhen Jiu. 2012 Apr;32(4):309-12.

56. Foster NE, Thomas E, Barlas P, Hill JC, Young J, Mason E, Hay EM. Acupuncture as an adjunct to exercise based physiotherapy for osteoarthritis of the knee: randomised controlled trial. BMJ. 2007 Sep 1;335(7617):436. doi: 10.1136/bmj.39280.509803.BE. Epub 2007 Aug 15.

57. CAI Guofeng, CAI Guoliang, ZHUANG Zhe, et al. Efficacy of millifire acupuncture combined with exercise therapy in the treatment of early knee osteoarthritis and its effect on serum NO[J]. Clinical Journal of Acupuncture,2020,36(7):20-25. DOI:10.3969/j.issn.1005-0779.2020.07.007.

58. Han Songmin. Clinical observation of the treatment of knee osteoarthritis by using the meridian remote extraction method combined with local meridian acupoints[D].Heilongjiang University of Traditional Chinese Medicine,2017.

59. Li Danhui. Clinical efficacy of muscle strength training combined with acupuncture in the treatment of knee osteoarthritis[D].Nanjing University of Chinese Medicine,2018.

60. Ren Hetang, Ding Wen, Qi Jingdong. Application effect of traditional Chinese medicine acupuncture combined with rehabilitation training in patients with early knee osteoarthritis[J]. China Health & Health,2022,40(15):31-33. DOI:10.3969/j.issn.1009-8011.2022.15.010.

61. XU Wei, SUN Dan, CHEN Na, et al. Efficacy of needle insertion analgesia combined with rehabilitation training on dysfunction of knee osteoarthritis[J]. Chinese Journal of General Practice,2017,15(12):2141-2144. DOI:10.16766/j.cnki.issn.1674-4152.2017.12.041.

62. Zhong Momo. Clinical study of Qihuang acupuncture in the treatment of knee osteoarthritis[D]. Guangdong:Guangzhou University of Chinese Medicine,2020.

63. Liu Guiping. Efficacy of electroacupuncture combined with exercise in the treatment of KOA[D]. Guangdong:Guangzhou University of Chinese Medicine,2016.

64. Tu JF, Yang JW, Shi GX, et al.Efficacy of Intensive Acupuncture Versus Sham Acupuncture in Knee Osteoarthritis: A Randomized Controlled Trial. Arthritis Rheumatol. 2021 Mar;73(3):448-458. doi: 10.1002/art.41584. Epub 2021 Jan 15.

65. Wang Mingjie, Wang Yueliang, Zhou Xuelong, et al. Clinical study of Yao Yi oil acupuncture exercise acupuncture in the treatment of knee arthritis with damp heat obstruction[J].Chinese Journal of Traditional Chinese Medicine,2021,36(08):5087-5091.

66. Chen Yiling. Clinical efficacy of squat training combined with acupuncture in the treatment of knee osteoarthritis[D].Fujian University of Chinese Medicine,2020.DOI:10.27021/d.cnki.gfjzc.

2020.000188.

67. WANG Qianbin, XIE Xuerong, HOU Meijin, et al. Clinical study on the effect of electricity on lower limb dynamics during ladder climbing in patients with knee osteoarthritis[J].Chinese Journal of Acupuncture,2017,37(10):1027-1034.DOI:10.13703/j.0255-2930.2017.10.001.

68. CHEN Gang,GU Ruixin,XU Dandan. Application of electroacupuncture in rehabilitation after total knee replacement[J]. Chinese Journal of Acupuncture,2012,32(4):309-312.

69. ZHANG Zhiqi, HU Jinlu, XIE Hui, et al. Clinical observation of 30 cases of knee osteoarthritis treated by bare hand impact therapy combined with local acupuncture[J].Hunan Journal of Traditional Chinese Medicine,2020,36(11):78-80.DOI:10.16808/j.cnki.issn1003-7705.2020.11.029.

70. Lin Hong. Study and analysis on the effect and recurrence of knee osteoarthritis combined with rehabilitation training combined with acupuncture and moxibustion of traditional Chinese medicine[J].Journal of Practical Cardio-Cerebrovascular Diseases,2020,28(S1):254-257.

71. ZHANG Zhanlei, SI Yan, MA Zou, et al. Effect of electroacupuncture combined with functional exercise on postoperative pain and early functional recovery of TKA in patients with KOA[J]. Sichuan Journal of Traditional Chinese Medicine,2020,38(11):193-196.

72. ZHAO Hui, XU Zhiguo. Clinical observation of "seven points of knee pain" in the treatment of mild to moderate knee osteoarthritis[J]. Sichuan Journal of Traditional Chinese Medicine,2020,38(2):191-194.

73. Yang Li, Wang Xiaoshan. Effects of out-of-hospital exercise rehabilitation on quality of life and muscle function in elderly patients with knee osteoarthritis[J]. Jilin Medical Journal,2022,43(11):3041-3044. DOI:10.3969/j.issn.1004-0412.2022.11.053.

**Low quality(n=28):**

1.Gan Piaoqin. Clinical observation of acupuncture with functional exercise for knee osteoarthritis[J].Clinical Journal of Traditional Chinese Medicine,2008(01):72-73. DOI:10.16448/j.cjtcm.2008.01.020.

2. ZHOU Zhongliang, YANG Yonghui, SUN Kui, et al. Clinical observation of acupuncture combined with exercise therapy in the treatment of knee osteoarthritis[J].Clinical Journal of Traditional Chinese Medicine,2007(05):481-482.DOI:10.16448/j.cjtcm.2007.05.066.

3. SHEN Tu Fengjun, LI Qiang, SHEN Aihong, et al. Clinical experience of distal limb acupoint taking combined with knee voluntary movement in the treatment of knee osteoarthritis[J]. Psychologist,2019,25(5):99-100.

4. WANG Junqing,ZHOU Xiaoyan. Clinical observation of 50 cases of acupuncture plus proprioceptive training in the treatment of knee osteoarthritis[J].Northwest National Defense Medical Journal,2012,33(05):572-573.DOI:10.16021/j.cnki.1007-8622.2012.05.010.

5. YANG Xiaolin,LIN Dong. Clinical observation of positive point and valley thorn combined with knee function training in the treatment of knee osteoarthritis[J]. Journal of Practical Chinese Medicine,2021,37(5):862-863.

6. JI Muqiang, OUYANG Minyi, HU Danxian, et al. Clinical observation of floating acupuncture therapy in the treatment of early knee osteoarthritis[J]. Chinese Folk Therapy,2021,29(7):35-37. DOI:10.19621/j.cnki.11-3555/r.2021.0715.

7. HUANG Douquan, NING Yingfeng, WANG Mingjian, et al. Baduanjin combined with electroacupuncture in the treatment of knee osteoarthritis: 35 cases[J]. Fujian Journal of Traditional Chinese Medicine,2021,52(12):42-44. DOI:10.3969/j.issn.1000-338X.2021.12.014.

8. CHEN Qingliang, XIE Jingxia, WANG Xiaoyan, et al. Clinical study of floating acupuncture combined with rehabilitation training in the treatment of knee osteoarthritis[J]. Shenzhen Journal of Integrated Traditional and Western Medicine,2021,31(10):73-75. DOI:10.16458/j.cnki.1007-0893.2021.10.033.

9. ZHANG Hao,QUE Qinghui. Wrist and ankle acupuncture combined with muscle strength training in the treatment of knee osteoarthritis in 40 cases[J]. Massage and Rehabilitation Medicine,2016,7(2):68-69.

10. Duo Zhao. Electroacupuncture combined with quadriceps functional exercise in the treatment of knee osteoarthritis[J]. Frontiers of Medicine,2015,5(35):199.

11. ZHANG Cuifen, CHENG Weifen, ZHANG Yeqiong, et al. Effect of exercise therapy combined with fire acupuncture in the treatment of knee osteoarthritis[J]. Massage and Rehabilitation Medicine,2012,3(5):197-198.

12. Hara Xingxing. Application of oblique meridian tendon combined with functional exercise in knee osteoarthritis[J]. Clinical Practice of Integrated Traditional Chinese and Western Medicine,2022,22(14):55-57. DOI:10.13638/j.issn.1671-4040.2022.14.016.

13. Xia Lingyue. Observation on the effect of Taiji acupuncture in the treatment of knee pain in Shaoyang people[J].Chinese Medical Guide,2019,17(24):195-196.DOI:10.15912/j.cnki.gocm.

2019.24.151.

14. Lang Boxu, Feng Chunyan, Fang Zhenyu. Treatment of knee osteoarthritis with acupuncture combined with functional exercise[J]. Clinical Journal of Acupuncture,2006,22(4):41-42. DOI:10.3969/j.issn.1005-0779.2006.04.024.

15. Li Yanhui. Treatment of proliferative knee arthritis in 50 cases with acupuncture combined with quadriceps exercise[J]. Chinese Journal of Clinical Medicine, 2003(60).

16. HE Yan,WU Di. Clinical effect of blade needle in the treatment of knee osteoarthritis[J]. Oriental Medicinal Diet, 2021(24):189.

17. Qi Yujun. Efficacy of warm acupuncture combined with rehabilitation training in the treatment of knee osteoarthritis[J]. Health Must Read,2019(34):287-288.

18. Wang Yanying. Acupuncture for the treatment of knee osteoarthritis[J].Chinese Folk Medicine,2009,17(06):7.DOI:10.19621/j.cnki.11-3555/r.2009.06.004.

19.He Shihua. Acupuncture treatment and rehabilitation training for knee osteoarthritis[J]. Medical Aesthetics and Cosmetology,2014(7):466-466.

20. SUN Ying. Clinical efficacy of warm compress of traditional Chinese medicine plus traditional Chinese medicine nursing in patients with osteoarthritis[J]. China Health and Nutrition, 2016(2):289-290.

21. Jin Xiaoping. Effect of acupuncture combined with rehabilitation training in the treatment of knee osteoarthritis[J]. Everyone Health,2017,11(10):46-47.

22. Ran Zhongjiang. Clinical effect of TCM rehabilitation in the treatment of acute onset of primary knee osteoarthritis[J]. Bifeet and Health,2017,26(16):72-73. DOI:10.19589/j.cnki.issn1004-6569.2017.16.072.

23. Dou Zhongwei, Hao Shu, Wei Ruifeng, et al. Clinical observation of acupuncture combined with exercise acupuncture plus acupoint application for paralysis in the treatment of knee osteoarthritis[J]. Inner Mongolia Journal of Traditional Chinese Medicine,2019,38(8):80-81.

24.LI Xiuhong, HE Yongchang, LUO Jianhua, et al. Effect of acupuncture exercise therapy combined with rehabilitation training in the intervention of middle-aged and elderly knee osteoarthritis in the community[J].China Contemporary Medicine,2017,24(19):74-77.

25. Zhao Tingting. Clinical study of acupuncture combined with exercise therapy in the treatment of knee osteoarthritis[J]. Chinese Journal of Disability Medicine,2021,29(9):52-53. DOI:10.13214/j.cnki.cjotadm.2021.09.033.

26. Niu Xiaojun. Acupuncture combined with rehabilitation training for the treatment of knee osteoarthritis: 78 cases[J].Chinese Journal of Ethnic and Folk Medicine,2010,19(17):187. DOI:10.3969/j.issn.1007-8517.2010.17.144.

27. CAI Yu, TANG Weidong, CHEN Hong, et al. Clinical study of acupuncture exercise therapy in the treatment of knee osteoarthritis[J]. Guangming Journal of Traditional Chinese Medicine,2017,32(9):1306-1308. DOI:10.3969/j.issn.1003-8914.2017.09.038.

28. Wang Lijie. Clinical effect of acupuncture combined with rehabilitation training in the treatment of knee arthritis in the elderly[J]. Journal of Continuing Medical Education,2021,35(9):166-167. DOI:10.3969/j.issn.1004-6763.2021.09.089.

**Others(n=62):**

**（1）The experimental group was combined with treatments other than the control group(n=38)**

1. Zhang T, Liu H, Li H, He S, Xiao L, Qin T, Xu WL. Effect of Early Electroacupuncture Combined with Enhanced Recovery after Surgery (ERAS) on Pain Perception and Dysfunction in Patients after Total Knee Arthroplasty (TKA). Biomed Res Int. 2022 May 9;2022:6560816. doi: 10.1155/2022/6560816.

2.SU Peipei, LI Qian, CHENG Xiaokang. Effects of acupuncture and electromagnetic wave irradiation combined with rehabilitation exercise training on rehabilitation effect and adverse reactions of knee osteoarthritis[J].Shezhi,2022,34(01):60-63. DOI:10.3969/j.issn.1001-5639.2022.01.016.

3. YANG Rong,MIAO Huiyu,HUANG Zhihui. Clinical observation of acupuncture combined with cupping exercise therapy in the treatment of knee osteoarthritis[J].Shanghai Journal of Acupuncture,2021,40(02):194-199.DOI:10.13460/j.issn.1005-0957.2021.02.0194.

4. HE Bo,LIU Yan,WANG Ping. Gait characteristics of radiofrequency acupuncture in the treatment of early and middle knee osteoarthritis under ultrasound visualization[J]. Tianjin Journal of Traditional Chinese Medicine,2022,39(5):604-610. DOI:10.11656/j.issn.1672-1519.2022.05.13.

5. Lin Shuyi. Clinical efficacy of Tai Chi combined with warm acupuncture in the treatment of early knee osteoarthritis [D]; Fujian University of Chinese Medicine,2021.

6.GU Xiaogang,CHEN Di,JIAO Anmei. Efficacy and prognosis of acupuncture and electromagnetic wave irradiation combined with rehabilitation exercise training on secondary prevention of knee osteoarthritis[J].Journal of Clinical Military Medicine,2021,49(04):453-454+457.DOI:10.16680/j.1671-3826.2021.04.37.

7. MA Rui, LIU Donghui. Efficacy of acupuncture combined with cupping exercise therapy in the treatment of knee osteoarthritis and its effect on bone metabolism indexes and inflammatory factors in patients[J].Shaanxi Journal of Traditional Chinese Medicine,2020,41(12):1813-1816. DOI:10.3969/j.issn.1000-7369.2020.12.036

8.KE Binxia. Clinical application of thermal moxibustion combined with acupuncture and rehabilitation training in the rehabilitation of knee osteoarthritis[J].Journal of Sino-Foreign Medical Research,2020,18(25):156-158.DOI:10.14033/j.cnki.cfmr.2020.25.063.

9. PAN Rong,CHEN Yiming. Clinical efficacy of electroacupuncture combined with functional exercise and external application of traditional Chinese medicine in the treatment of knee osteoarthritis[J]. Health Care Medical Research and Practice,2021,18(4):86-90. DOI:10.11986/j.issn.1673-873X.2021.04.019.

10. QIU Jianqing,LIU Shuru,LIN Qianlin,et al. Acupuncture combined with cupping exercise therapy for the treatment of Qi stasis knee osteoarthritis:a randomized controlled study[J].Chinese Journal of Acupuncture,2019,39(05):462-466.DOI:10.13703/j.0255-2930.2019.05.002.

11. FU Cong,FANG Zheng,ZHU Manhua. Application effect of thermal moxibustion combined with acupuncture and rehabilitation training in patients with knee osteoarthritis[J].China Contemporary Medicine,2019,26(21):138-140. DOI:10.3969/j.issn.1674-4721.2019.21.042.

12. WANG Gang,DONG Baoqiang. Multicenter randomized parallel-controlled study of merthenon acupuncture + massage stretching + eight-style knee functional rehabilitation exercise in the treatment of early and middle knee osteoarthritis (walking paralysis/wind paralysis, pain paralysis/chills) in northern cold land[J].Journal of Practical Chinese Internal Medicine,2018,32(06):61-65.DOI:10.13729/j.issn.1671-7813.z20170546.

13. Tian Ke. Randomized parallel-controlled study of acupuncture-Tuina massage-medicinal bath-functional exercise in elderly osteoarthritis (spleen deficiency and waterfall)[J].Journal of Practical Chinese Internal Medicine,2018,32(05):59-61.DOI:10.13729/j.issn.1671-7813.z20180038.

14. Cao Wenwen. Observation and nursing of warm acupuncture and tuina therapy in the treatment of knee osteoarthritis[J].Health Road,2018,17(03):227.

15. XU Cankang, HUANG Yanghua. Clinical efficacy of TCM directional permeation combined with acupuncture and exercise therapy in the treatment of knee osteoarthritis[J].Minimally Invasive Medicine,2017,12(05):709-711. DOI:10.11864/j.issn.

1673.2017.05.43.

16. ZHAO Weiwei, FU Peng, LIN Jin. Clinical observation of isometric contraction training of flexor and extensor muscles combined with external application of traditional Chinese medicine in the treatment of KOA based on muscle chain theory[J]. World Latest Medical Information Digest,2017(4):68-69.

17. LI Xiuhong, HE Yongchang, LUO Jianhua, et al. Effect of acupuncture exercise therapy combined with rehabilitation training in the intervention of middle-aged and elderly knee osteoarthritis in the community[J].China Contemporary Medicine,2017,24(19):74-77.

18. Zhang Kaiyong, Yang Yang, Shou Kun, et al. Efficacy of warm acupuncture combined with functional exercise in the treatment of knee osteoarthritis[J]. Journal of Acupuncture and Tuina Science,2016,14(06):412-415. DOI:10.1007/s11726-016-0959-6.

19. Zhang Hao. Effect of wrist and ankle needle combined with elastic band training on flexor and extensor muscle function and balance ability in knee osteoarthritis[D].Fujian University of Chinese Medicine,2017.

20.Chen Jiangbo, Feng Xianli. Clinical efficacy of acupuncture, thermal moxibustion combined with rehabilitation training in the treatment of knee osteoarthritis[J].World Journal of Integrated Western Medicine,2015,10(11):1577-1579+1588.DOI:10.13935/j.cnki.sjzx.151129.

21. Liu Ying, Jiang Xiaoli. Efficacy of physiotherapy, acupuncture and functional training in the treatment of knee osteoarthritis[J].Shaanxi Journal of Traditional Chinese Medicine,2014,35(08):1065-1067. DOI:10.3969/j.issn.1000-7369.2014.08.062.

22. Li Xuejiao. Clinical efficacy of acupuncture, heat-sensitive moxibustion combined with rehabilitation training in the treatment of knee osteoarthritis in 42 cases[J].Asia-Pacific Journal of Traditional Medicine,2014,10(06):98-99.

23.Zhang Yong. Triple therapy for the treatment of knee osteoarthritis in 30 cases[J].Modern Distance Education of Chinese Medicine,2013,11(22):76. DOI:10.3969/j.issn.1672-2779.2013.22.060.

24. Wang Di, Liu Huiqin. Triple therapy for the treatment of knee osteoarthritis in 50 cases[J].Chinese Folk Therapy,2013,21(06):44.DOI:10.19621/j.cnki.11-3555/r.2013.06.040.

25. QIU Ling, ZHENG Xu, ZHAI Jiali, et al. Effect of warm acupuncture combined with rehabilitation training on knee joint function of knee osteoarthritis in post-earthquake plate area[J].Sichuan Journal of Traditional Chinese Medicine,2013,31(05):126-129.

26. FAN Yuanzhi, GONG Li, YAN Juntao, et al. Efficacy of acupuncture and tuina combined with rehabilitation training in the treatment of knee osteoarthritis[J].Shanghai Journal of Acupuncture,2011,30(01):30-32. DOI:10.3969/j.issn.1005-0957.2011.01.030.

27. CHEN Wei,LI Jing,LIU Guizhen,ZHANG Fuqing. Clinical study of warm acupuncture combined with isokinetic muscle strength training in the treatment of knee osteoarthritis[J].Acupuncture and Tuina Medicine:English Edition,2022,20(3):221-228.

28. ZHAO Hui, XU Zhiguo. Clinical observation of "seven points of knee pain" in the treatment of mild to moderate knee osteoarthritis[J]. Sichuan Journal of Traditional Chinese Medicine,2020,38(2):191-194.

29. SHEN Hai, HE Chunjiang, LUO Xiaobing, et al. Effect of hip-knee combined acupuncture on lower limb motor function in patients with early and middle knee osteoarthritis[J].Journal of Traditional Chinese Medicine,2018,30(12):4-8.

30. Zhang Junping. Comparative analysis of the clinical efficacy of rehabilitation training after electroacupuncture plus infrared local irradiation and rehabilitation training alone in the treatment of knee osteoarthritis[J]. Chinese Journal of Disability Medicine, 2015(14):139-140. DOI:10.13214/j.cnki.cjotadm.2015.14.105.

31.Zhang Xiaoyan, Liu Yuanming. Application of electroacupuncture plus infrared local irradiation to rehabilitation of knee osteoarthritis[J]. Medical Information,2014(16):617-617. DOI:10.3969/j.issn.1006-1959.2014.16.829.

32. Guo JM, Xiao Y, Cai TY, Wang JH, Li BL, Huang LL, Mao X, Lai XQ, Zhu YJ, Zhang YQ, Chen SQ, Su YX. Chinese Medicine Involving Triple Rehabilitation Therapy for Knee Osteoarthritis in 696 Outpatients: A Multi-Center, Randomized Controlled Trial. Chin J Integr Med. 2021 Oct;27(10):729-736. doi: 10.1007/s11655-021-3488-6. Epub 2021 Mar 12.

33. LI Jinqiu, XIE Liyan, ZHAO Rongxun, et al. Effects of warm acupuncture combined with rehabilitation training on pain and quality of life in patients with knee osteoarthritis[J]. Modern Chinese Medicine,2020,40(4):65-67. DOI:10.13424/j.cnki.mtcm.2020.04.016.

34. Wu Ping. Clinical effect of warm acupuncture combined with exercise therapy in patients with knee osteoarthritis[J].Medical Equipment,2017,30(02):22-23.

35.Wang Baocheng. Efficacy of warm acupuncture combined with exercise therapy on degenerative knee arthritis[J]. Clinical Research of Traditional Chinese Medicine,2014(28):35-36. DOI:10.3969/j.issn.1674-7860.2014.28.016.

36. Su Guolong, Ge Weifeng, Zhong Cankun, et al. Randomized parallel-controlled study of electroacupuncture, self-massage combined with strength training in the treatment of knee osteoarthritis[J].Journal of Practical Chinese Internal Medicine,2013,27(04):24-26.

37. QIU Ling, ZHAI Jiali, LIU Di, et al. Clinical study of warm acupuncture combined with rehabilitation training in the treatment of knee osteoarthritis in the post-earthquake plate atrial area[J].Journal of Practical Medicine,2011,27(12):2261-2263.

38. WANG Shudong,GUO Haiqing,WANG Huawei. Clinical study of acupuncture combined with joint loosening in the treatment of knee osteoarthritis[J].Chinese Journal of Traditional Chinese Medicine,2021,39(08):179-182.DOI:10.13193/j.issn.1673-7717.2021.08.042.

**（2）Both intervention methods were acupuncture + active exercise training(n=15)**

1. XUE Yingyu. Clinical efficacy of electroacupuncture combined with quadriceps functional exercise in the treatment of knee osteoarthritis[D].Fujian University of Chinese Medicine,2022.DOI:10.27021/d.cnki.gfjzc.2022.000131.

2.LI Xiaomei, ZHANG Weixiong, JIANG Cai,et al. Clinical observation of acupuncture combined with acupuncture in the acute phase of early knee osteoarthritis[J].Trauma and Emergency Electronic Journal,2021,9(03):161-164.DOI:10.16746/j.cnki.11-9332/r.2021.03.0

07.

3. LI Yingchun,ZHU Junchen,WANG Chao,et al. Clinical observation of acupuncture release combined with quadriceps exercise in the treatment of varus knee osteoarthritis[J].Journal of Neck and Low Back Pain,2020,41(01):65-67. DOI:10.3969/j.issn.1005-7234.2020.01.019.

4. GUO Jiayi,LI Feng,FAN Yiming,et al. Effect of oblique meridian tendon method combined with exercise therapy on joint function in patients with knee osteoarthritis[J].Chinese Journal of Traditional Chinese Medicine,2019,34(10):4988-4992.

5. Wen Hongyuan, Ge Minen, Dai Yiqi, et al. Clinical observation of oblique pricking meridian tendon combined with exercise in the treatment of knee osteoarthritis[J].Research of Integrated Traditional Chinese and Western Medicine,2022,14(04):250-252. DOI:10.3969/j.issn.1674-4616.2022.04.009.

6. MA Jiuli, CHEN Nanping, ZHANG Yumei. Clinical observation of acupuncture combined with quadriceps training in the treatment of knee osteoarthritis[J].Modern Distance Education of Chinese Medicine,2022,20(06):116-118. DOI:10.3969/j.issn.1672-2779.2022.06.044.

7. Zhao Tingting. Clinical study of acupuncture combined with exercise therapy in the treatment of knee osteoarthritis[J]. Chinese Journal of Disability Medicine,2021,29(9):52-53. DOI:10.13214/j.cnki.cjotadm.2021.09.033.

8. HE Wei, MA Hongxi. Clinical observation of transacupuncture combined with quadriceps traction training in the treatment of knee osteoarthritis[J]. Journal of Practical Chinese Medicine,2019,35(6):740-741.

9. Chen Xiaohui. Efficacy of floating needle combined with quadriceps training in the treatment of knee osteoarthritis[D]. Guangdong:Guangzhou University of Chinese Medicine,2019.

10. LI Kang, LU Jin, TIAN Yali, et al. Clinical observation of floating needle combined with reperfusion activity in the treatment of knee osteoarthritis[J]. Journal of Hunan University of Chinese Medicine,2018,38(3):315-319. DOI:10.3969/j.issn.1674-070X.2018.03.020.

11. LI Xiaoyan,LIU Erlin,GAO Weibin. Clinical observation of 60 cases of knee osteoarthritis treated by electroacupuncture Futu and Liangqiu acupoint combined with quadriceps muscle strength training[J]. Chinese Journal of Disability Medicine, 2014(8):47-48.

12. LIU Yuan. Clinical study of balanced acupuncture plus functional exercise in the treatment of knee osteoarthritis[D]. Henan University of Chinese Medicine,2011.

13.Han Senning. Clinical efficacy of electroacupuncture in the treatment of knee osteoarthritis[J]. Clinical Practice of Integrated Traditional Chinese and Western Medicine,2019,19(4):25-27. DOI:10.13638/j.issn.1671-4040.2019.04.012.

14. Hengdun Qian, Zhou Satellite. Clinical study of floating needle in the treatment of knee osteoarthritis[J]. Journal of Practical Chinese Medicine,2018,34(1):100-101. DOI:10.3969/j.issn.1004-2814.2018.01.074.

15.WEI Xuqiang,ZHANG Deqing. Clinical efficacy of acupuncture combined with exercise therapy in the treatment of knee osteoarthritis[J]. Asia-Pacific Journal of Traditional Medicine,2014,10(9):80-83.

**（3）Review or conference abstract (n=9)**

1. ZHANG Lihua,LI Jiani,ZHANG Xiaohan,et al. Based on the theory of "bone, tendon and muscle" in the Yellow Emperor's Internal Canon, acupuncture combined with exercise therapy in the treatment of knee osteoarthritis[J].Chinese Journal of Traditional Chinese Medicine,2022,37(07):3965-3968.

2. YU Caidan,WEI Rong,HU Yuanyuan,et al. Clinical observation of acupuncture exercise therapy in the treatment of knee osteoarthritis[J].Hubei Journal of Traditional Chinese Medicine,2019,41(08):56-58.

3. XIA Qiufang,SUN Yanan,LI Haiyan,et al. Observation on the efficacy of acupuncture with functional exercise in the treatment of knee osteoarthritis[C]//Proceedings of the 2017 World Acupuncture Conference and 2017 Chinese Acupuncture and Moxibustion Society Annual Conference. 2017:2.

4. Gan Puqin, Sun Kui, Zhang Hong. Clinical observation and nursing of acupuncture with functional exercise for the treatment of knee osteoarthritis[C]//China Association of Chinese Medicine, Nursing Branch of Chinese Association of Chinese Medicine. Proceedings of the 2nd 2nd Conference on Chinese Medicine Nursing Academic Exchange. [Publisher unknown], 2007:3.

5. Chen Shurong. Clinical observation of acupuncture combined with exercise therapy in the treatment of knee osteoarthritis[C]//Chinese Medical Association Physical Medicine and Rehabilitation Branch. Proceedings of the 9th National Conference on Physical Medicine and Rehabilitation of Chinese Medical Association. [Publisher unknown], 2007:2.

6. Zhang Zhanbo, Evaluation of the efficacy of acupuncture exercise therapy in the treatment of knee osteoarthritis. Affiliated Hospital of North China University of Science and Technology,Hebei Province,2015-12-30.

7. LIU Xin, LUO Jiaqi, HU Wenjiao, et al. Research progress of external treatment of traditional Chinese medicine in the treatment of knee osteoarthritis[J]. Chinese Ethnic and Folk Medicine,2022,31(22):64-68.

8. ZHOU Yong. Acupuncture combined with rehabilitation exercise in the treatment of knee osteoarthritis in 42 cases[C]. The Second Academic Conference of the Acupuncture and Moxibustion Rehabilitation Professional Committee of the Chinese Acupuncture and Moxibustion Society, the First Academic Conference of the Rehabilitation Medicine Professional Committee of the 3rd Shandong Association of Integrative Medicine, and the First Academic Conference of the Rehabilitation Nursing Professional Committee of the 1st Shandong Nursing Association. 2014:368-370.

9. DONG Xinchun,SU Bin. Clinical efficacy analysis of acupuncture combined with functional training in the treatment of knee osteoarthritis in the elderly[C]. Proceedings of the 10th Annual Conference of Rehabilitation Therapy of Chinese Association of Rehabilitation Medicine in 2013. 2013:673-675.
