# Supplementary material for: Efficacy of acupuncture combined with active exercise training in improving pain and function of knee osteoarthritis individuals: a systematic review and meta-analysis
Source: J Orthop Surg Res. 2023 Dec 2;18:921. doi: 10.1186/s13018-023-04403-2 (PMC10693122; doi:10.1186/s13018-023-04403-2)
Supplement: Supplementary file 4 — Additional file 4: Quality of evidence per outcome from selected studies by the GRADE approach. [file 13018_2023_4403_MOESM4_ESM.docx]

**Quality of evidence per outcome from selected studies by the GRADE approach**

| **Quality assessment** | | | | | | | **Number of patients** | | **Effect** | | **Quality** | **Importance** |
| --- | --- | --- | --- | --- | --- | --- | --- | --- | --- | --- | --- | --- |
|  |  |  |  |  |  |  |  |  |  |  |  |  |
| **Number of studies** | **Design** | **Risk of bias** | **Inconsistency** | **Indirectness** | **Imprecision** | **Other considerations** | **EG** | **CG** | **Relative (95% CI)** | **Absolute**  **(95% CI)** |  |  |
| **Total efficiency rate (Combination group versus Acupuncture group)** | | | | | | | | | | | | |
| 7 | RCT | serious | no serious inconsistency | no serious indirectness | no serious imprecision | none | 218 | 223 | RR 1.13  (1.05 to 1.22) | - | ⊕⊕⊕O MODERATE | CRITICAL |
| **VAS (Combination group versus Acupuncture group)** | | | | | | | | | | | | |
| 7 | RCT | serious | serious | no serious indirectness | no serious imprecision | none | 218 | 223 | - | MD -0.74(-1.04 to -0.43) | ⊕⊕OO LOW | IMPORTANT |
| **WOMAC total score (Combination group versus Acupuncture group)** | | | | | | | | | | | | |
| 7 | RCT | serious | serious | no serious indirectness | no serious imprecision | none | 218 | 223 | - | MD -6.97(-10.74 to -3.19) | ⊕⊕OO LOW | IMPORTANT |
| **WOMAC-dysfunction (Combination group versus Acupuncture group)** | | | | | | | | | | | | |
| 3 | RCT | serious | serious | no serious indirectness | serious | none | 91 | 95 | - | MD -7.69(-18.34 to 2.96) | ÅOOO VERY LOW | CRITICAL |
| **WOMAC-stiffness (Combination group versus Acupuncture group)** | | | | | | | | | | | | |
| 3 | RCT | serious | serious | no serious indirectness | serious | none | 91 | 95 | - | MD -1.08(-2.19 to 0.02) | ÅOOO VERY LOW | CRITICAL |
| **WOMAC-pain (Combination group versus Acupuncture group)** | | | | | | | | | | | | |
| 3 | RCT | serious | serious | no serious indirectness | serious | none | 91 | 95 | - | MD -1.08(-2.57 to 0.40) | ÅOOO VERY LOW | CRITICAL |
| **ROM (Combination group versus Acupuncture group)** | | | | | | | | | | | | |
| 2 | RCT | serious | no serious inconsistency | no serious indirectness | serious | none | 59 | 60 | - | MD 6.21(2.37 to 10.04) | ⊕⊕OO LOW | IMPORTANT |
| **Total efficiency rate (Combination group versus Non-acupuncture group)** | | | | | | | | | | | | |
| 5 | RCT | serious | no serious inconsistency | no serious indirectness | serious | none | 161 | 160 | RR 1.31  (1.18 to 1.47) | - | ⊕⊕OO LOW | IMPORTANT |
| **VAS (Combination group versus Non-acupuncture group)** | | | | | | | | | | | | |
| 5 | RCT | serious | serious | no serious indirectness | serious | none | 161 | 160 | - | MD -1.42(-1.85 to -1.00) | ÅOOO VERY LOW | CRITICAL |
| **WOMAC total score(Combination group versus Non-acupuncture group)** | | | | | | | | | | | | |
| 4 | RCT | serious | serious | no serious indirectness | serious | none | 129 | 128 | - | MD -7.05(-11.43 to -2.66) | ÅOOO VERY LOW | CRITICAL |
| **WOMAC-dysfunction (Combination group versus Non-acupuncture group)** | | | | | | | | | | | | |
| 2 | RCT | serious | no serious inconsistency | no serious indirectness | serious | none | 69 | 68 | - | MD -5.34(-7.81 to -2.87) | ⊕⊕OO LOW | IMPORTANT |
| **WOMAC-stiffness (Combination group versus Non-acupuncture group)** | | | | | | | | | | | | |
| 2 | RCT | serious | no serious inconsistency | no serious indirectness | serious | none | 69 | 68 | - | MD -0.39(-0.73 to -0.06) | ⊕⊕OO LOW | IMPORTANT |
| **WOMAC-pain (Combination group versus Non-acupuncture group)** | | | | | | | | | | | | |
| 2 | RCT | serious | no serious inconsistency | no serious indirectness | serious | none | 69 | 68 | - | MD -1.43(-2.13 to -0.73) | ⊕⊕OO LOW | IMPORTANT |

Abbreviations: RCT, randomised controlled trial; EG, experimental group; CG, control group; CI, confidence interval; RR, risk ratio; MD, mean difference;
